# Discovery of circadian rhythm-related hub genes in acute myocardial infarction: A two-sample Mendelian randomization study

**DOI:** 10.1097/MD.0000000000047228

**Published:** 2026-01-16

**Authors:** Song Peng, Yan Leng, Man-Hua Chen

**Affiliations:** a Department of Cardiology, The Central Hospital of Wuhan, Tongji Medical College, Huazhong University of Science and Technology, Wuhan, China; b Key Laboratory for Molecular Diagnosis of Hubei Province, The Central Hospital of Wuhan, Tongji Medical College, Huazhong University of Science and Technology, Wuhan, China; c Department of Anesthesiology, Renmin Hospital of Wuhan University, Wuhan, China.

**Keywords:** acute myocardial infarction, circadian rhythm, Mendelian randomization, molecular regulatory network

## Abstract

Circadian rhythms have been reported in a variety of physiological processes that may influence cardiovascular disease, while little is known about the effects of circadian rhythm-related genes (CRRGs) on acute myocardial infarction (AMI). The genome-wide association study (GWAS) data of AMI (ukb-a-533) and expression quantitative trait loci (eQTL) data of CRRGs were downloaded from the integrative epidemiology unit Open GWAS database. The relationship between the CRRGs and AMI was assessed by the two-sample Mendelian randomization (TSMR) analysis. The hub genes that could directly affect AMI were identified based on the inverse variance weighted (IVW) algorithms. Subsequently, the TSMR results were evaluated via sensitivity analyses and MR-Steiger filtering. Then, the expression in immune cells and tissues was predicted from the Human Protein Atlas and Genotype-Tissue Expression databases. Finally, the molecular regulatory networks were generated based on the hub genes. In TSMR results, NR1H3 (IVW: odds ratio (OR) = 1.0009, 95% confidence interval (CI) = 1.0005–1.0013), SREBF1 (IVW: OR = 1.0015, 95% CI = 1.0007–1.0022), SIRT1 (IVW: OR = 1.0007, 95% CI = 1.0001–1.0013), and HIF1A (IVW: OR = 1.0022, 95% CI = 1.0004–1.0039) were risk factors for AMI patients, while NCOA1 (IVW: OR = 0.9984, 95% CI = 0.9969–0.9998) was a protective factor for AMI patients (*P* < .05). Importantly, the 5 hub genes could affect AMI occurrence in one direction. The expression levels of HIF1A, NCOA1, and SREBF1 were highest in neutrophils than the other immune cells. Also, HIF1A and SREBF1 had higher expression in the heart (left ventricle and atrial appendage) and artery (aorta, tibial, and coronary). Moreover, the transcription factor, NFKB1, might regulate the hub genes except for NCOA1. Generally, 4 risk genes (NR1H3, SREBF1, SIRT1, and HIF1A) and 1 protective gene (NCOA1) associated with circadian rhythm for AMI patients were identified, providing new insights into the diagnosis and treatment of AMI.

## 1. Introduction

Over the past 3 decades, with improvements in living standards, the aging population, and changes in dietary habits (such as increased intake of high-sugar and high-fat foods, and decreased consumption of fruits and vegetables), the number of global patients with coronary heart disease (CHD) has been steadily increasing. CHD is a major cause of death worldwide, particularly acute myocardial infarction (AMI), a form of CHD characterized by the irreversible necrosis of cardiac myocytes due to acute ischemia.^[1]^ In the United States alone, approximately 1.7 million individuals are diagnosed with myocardial infarction each year, and over 8 million are hospitalized due to symptoms associated with AMI.^[2]^ Meanwhile, in China, alongside rapid socioeconomic transformation, the age-standardized incidence rate of AMI has also shown a notable upward trend over recent decades. Although the incidence of AMI varies across populations from different regions, there is still a research gap regarding the mechanisms of circadian rhythm genes in the pathogenesis of AMI in European populations. This study focuses on European populations and aims to explore the fundamental role of circadian rhythm-related genes (CRRGs) in the onset of AMI. Several risk factors contribute to the onset of AMI, including smoking, obesity, hypertension, hyperlipidemia, diabetes, and a family history of the disease. Atherosclerosis serves as the underlying cause, with mechanisms such as cholesterol metabolism disturbances, endothelial cell damage, smooth muscle cell proliferation, thrombosis, and inflammation leading to the development of AMI.^[3]^ A decline in left ventricular ejection fraction and the occurrence of malignant arrhythmias can severely impact a patient’s quality of life following an AMI.^[4]^ In recent years, the incidence and mortality rates of AMI are increasing, especially the young man (<40 years old), with their incidence surging from 1.20% in 2014 to 1.43% in 2017,^[5]^ The escalating prevalence and high mortality rate of AMI in this demographic have imposed a significant health and economic burden on society,^[6]^ which urges researchers to develop novel and more effective therapeutic strategies. Therefore, it is critical to develop effective strategies for the early prevention, diagnosis, and treatment of AMI.

It is well-known that human activities in contemporary society, such as extended working hours, unhealthy lifestyle habits (such as prolonged electronic device usage) and irregular sleep schedules disrupting normal circadian rhythms, leading to poor sleep quality and hormonal imbalances, have shifted in relation to day-night rhythms. The circadian system plays a crucial role in regulating internal functions of the body according to environmental cues. Disruption of the circadian rhythm, known as chronodisruption, can lead to changes in quality of life and contribute to a range of diseases, from mental health disorders to autoimmune conditions.^[7]^ It also affects the cardiovascular (CV) system by inducing diurnal variations in heart rate, blood pressure, cardiac output, and endothelial function, among other physiological parameters.^[8]^ Various CV events, including CHD, arrhythmias, AMI, and sudden cardiac death, also exhibit diurnal variation.^[9]^ Clock genes, such as BMAL1 and Per2, are associated with both the physiology and pathophysiology of the CV system.^[10]^ Recent studies in mice have demonstrated that myocardial ischemia/reperfusion injury follows a circadian pattern.^[11]^ Additionally, the incidence of AMI is consistently higher during the transition from dark to light (6:00 am to noon), with Fournier^[12]^ and his team finding that myocardial infarction sizes are larger in patients whose symptoms begin between 12:00 am and 5:59 am This suggests that diurnal rhythms could be closely linked to the onset of myocardial infarction. However, the relationship between circadian rhythm genes and AMI has not yet been systematically explored.

Mendelian randomization (MR) is a type of instrumental variable analysis that can be used to test causal hypotheses in non-experimental data. In MR analysis, genetic variants serve as instrumental variables for the putative risk factor. MR takes advantage of the random allocation of genetic variants at conception, making it less prone to confounding and reverse causality compared to traditional observational studies.^[13]^ To produce valid results in MR analysis, 3 key assumptions must be met: the genetic variant must be strongly associated with the risk factor; the genetic variant must not be associated with any known or unknown confounders; and the genetic variant must influence the outcome solely through the risk factor and not via any direct causal pathways.^[14]^ Recent studies utilizing MR analysis have revealed no interactive effects among sleep traits on the risk of AMI.^[15]^ However, combinations of all sleep traits were found to increase AMI risk, except in individuals with longer sleep duration. This suggests that the primary effects of sleep traits on AMI are likely independent of each other.^[15]^ In a two-sample Mendelian randomization (TSMR) study, the associations between the genetic variant and the risk factor, as well as between the genetic variant and the outcome, come from independent study populations. The use of large-scale genome-wide association study (GWAS) consortia data enhances statistical power, as it provides access to existing summarized data. However, research on the interaction between CRRGs and AMI using MR remains limited. Notably, the growing availability of genetic association resources offers a valuable opportunity to explore the causal effects of various risk factors on AMI.

Against this backdrop, Current research on the association between CRRGs and AMI is still insufficient. Based on large-scale GWAS data from European populations, this study employs a TSMR approach to investigate the causal relationship between CRRGs and AMI. It aims to identify hub genes and perform functional enrichment analysis, chromosomal and subcellular localization analysis, expression analysis, and the construction of molecular regulatory networks for these hub genes. The findings will lay the groundwork for elucidating the core role and regulatory pathways of CRRGs in the pathological progression of AMI, thereby providing theoretical support and research directions for optimizing clinical prevention and treatment strategies for AMI and advancing the development of personalized diagnosis and therapy.

## 2. Materials and methods

### 2.1. Data acquisition

The 261 CRRGs were collected from the molecular signatures database (MSigDB; https://www.gsea-msigdb.org/gsea/msigdb), with “circadian rhythm” as the keyword. For TSMR analysis, based on the keyword “AMI,” the ukb-a-533 was downloaded from the Integrative Epidemiology Unit (IEU) Open GWAS database (https://gwas.mrcieu.ac.uk/). The ukb-a-533 dataset consisted of 3927 AMI European patients and 333,272 European healthy controls, and they were all European. There were 10,894,596 single nucleotide polymorphisms (SNPs) in the ukb-a-533 dataset. Also, the expression quantitative trait loci (eQTL) data of CRRGs were scoured from the IEU Open GWAS database. GWAS summary statistics for AMI were acquired from prior research. Therefore, additional ethical approval was not required.

### 2.2. Instrument variables (IVs) selection

Here, the TwoSampleMR (v. 0.5.8) package^[16]^ was applied for the TSMR analysis. The CRRGs (eQTL data) were exposure factors and the AMI (ukb-a-533) was an outcome variable in this study. To obtain eligible instrumental variables (IVs), extract_instruments function was employed to extract exposure factors, outcome, and screen IVs. Screening criteria were as follows: With parameter set as *P* < 5 × 10^−8^, IVs notably associated with exposure factors were searched; With clump = TRUE, R2 = 0.001, and kb = 200, IVs with linkage disequilibrium were excluded to ensure genetic independence of IVs; based on outcome GWAS data and previously screened IVs, with proxies = TRUE and rsq = 0.8, IVs notably associated with outcome were removed; exposure factors with *F* (statistic) values >10 for SNPs (to exclude weak instruments with potential bias) and number of SNPs ≥ 3 (to ensure sufficient statistical power of IVs) were retained for subsequent analysis. The *F*-values formula is as follows: *F*=β2/SE2, where β is the effect size of the allele and SE is the standard error. Then, the harmonise_data function was applied to standardize the effect alleles and effect sizes, and exposure factors – IVs – outcome were matched.^[17]^

In TSMR analysis, MR Egger,^[18]^ Weighted median,^[19]^ Inverse variance weighted (IVW),^[20]^ Simple mode,^[16]^ and Weighted mode^[21]^ tests were used to explore the causal correlation between the CRRGs and the AMI using the mr function (*P* < .05). The IVW test was considered the principal method. Then, the TSMR results were expressed as odds ratio (OR) with a 95% confidence interval (CI). OR < 1 indicated that the exposure factor was a protective factor for AMI patients, whereas OR > 1 was a risk factor. In addition, to ensure the reliability of the statistical results, the p.adjust function from the base package (v 4.3.3) (https://www.R-project.org/) was used, and the false discovery rate method was adopted for multiple test correction.

In order to determine the correlation between exposure factors and outcomes, the mr_scatter_plot function was used to plot the correlation scatter plots by combining the SNP-exposure factors effect and the SNP-outcomes effect. A positive slope of the line indicates a risk factor and a negative slope of the line indicates a safety factor. When an intercept is present, the presence of confounding factors is implied. To determine the diagnostic efficacy of each SNP locus for outcomes, the mr_forest_plot function was used to create a forest plot to show the diagnostic efficacy of each SNP locus for outcomes by each exposure factor. The SNP points on the left side of the dotted line are the safety factors, and the SNP points on the right side of the dotted line are the risk factors. The red line is the overall effect of the IVW model. To determine whether the analysis of the outcome variable by each SNP serving as an exposure factor was randomized and whether the MR analysis conformed to Mendel second law of random assortment, a funnel plot was generated using the mr_funnel_plot function for randomization assessment. If the SNPs were symmetrically distributed on both sides of the funnel plot, it indicated that the MR analysis conformed to Mendel’s second law of random assortment.

### 2.3. Sensitivity analyses and MR-Steiger filtering

To evaluate the validity of the TSMR results, sensitivity analyses were performed, including heterogeneity, pleiotropy, and leave-one-out (LOO) sensitivity tests.^[22]^ Using the mr_heterogeneity function, Cochran *Q* test was applied to detect the heterogeneity between the CRRGs and AMI datasets (*Q* > 0.05). When *Q*-pvalue > .05, it indicated no heterogeneity, and the fixed-effects of IVW algorithm was used for the MR analysis. When the *Q*-pvalue < .05, there was heterogeneity, and the random-effects of IVW algorithm was used for the MR analysis, while *Q*-pvalue > .05 indicated that there was no heterogeneity. The pleiotropy test was used to determine whether there were confounding factors in the study by the mr_pleiotropy_test function and MR-PRESSO (v. 1.0) package (*P* > .05) [Verbanck M (2017). _MRPRESSO: Performs the MR Pleiotropy RESidual Sum and Outlier (MR-PRESSO) test. R package version 1.0.]. In the LOO sensitivity test, individual SNP was gradually included, the meta effect of the remaining SNPs was calculated, and whether the results significantly changed was observed via the mr_leaveoneout function. Last, MR-Steiger filtering was applied to confirm whether exposure factors affected AMI in only one direction (correct_causal_direction = TRUE, *P* < .05). Overall, the genes passed above analyses were termed hub genes.

### 2.4. Functional enrichment analysis

To explore more functions of hub genes, the gene ontology (GO) and Kyoto Encyclopedia of Genes and Genomes (KEGG) enrichment analysis were subjected by the clusterProfiler (v. 4.4.4) and org. Hs.e.g..db (v. 3.17.0) packages.^[23,24]^ Especially, the GO analysis included biological process (blood pressure), molecular function, and cellular component items. Finally, the GO items and KEGG pathways related to hub genes were obtained (*P* < .05). Furthermore, the above results were visualized using the enrichplot (v. 1.18.3) package.^[23]^

### 2.5. Chromosome and subcellular localizations

Here, with the intention of exploring the location of hub genes on the chromosomes, the chromosome localization of hub genes was analyzed via the RCircos (v. 1.2.0) package.^[25]^ Subsequently, to explore hub gene-related subcellular organelles the fasta files of hub gene sequences were downloaded from the National Center for Biotechnology Information (https://www.ncbi.nlm.nih.gov/) database. After uploading to the mRNALocater database,^[25]^ the subcellular localizations of hub genes were predicted.

### 2.6. The expression level of hub genes in immune cells and tissues

To obtain hub gene-associated immune cells and tissues, the expression levels of hub genes in immune cells were predicted from the Human Protein Atlas (https://www.proteinatlas.org/) database. Also, the expression levels of hub genes in distinct tissues were predicted from the Genotype-Tissue Expression (https://www.gtexportal.org/home/) database.

### 2.7. Transcription factor (TF)-mRNA network and gene–gene interaction (GGI) network

To delve into TFs that target-regulated hub genes, potential TFs that regulated hub genes were predicted based on NetworkAnalyst (https://www.networkanalyst.ca/). Moreover, to investigate the functional status of the hub genes and their associated genes, the top 20 genes related to hub genes were predicted based on the GeneMANIA (http://www.genemania.org/) database. The TF-mRNA and gene–gene interaction (GGI) networks were established and visualized utilizing the Cytoscape (v. 3.9.1) package.^[26]^

### 2.8. Statistical analysis

Statistical computations were executed via the R statistical software (v. 4.2.2). TSMR analysis was performed using the TwoSampleMR (v. 0.5.8) package, and statistical significance was determined at a significance level of *P* < .05.

## 3. Results

### 3.1. A total of 5 hub genes could directly affect AMI occurrence

**Figure 1.**
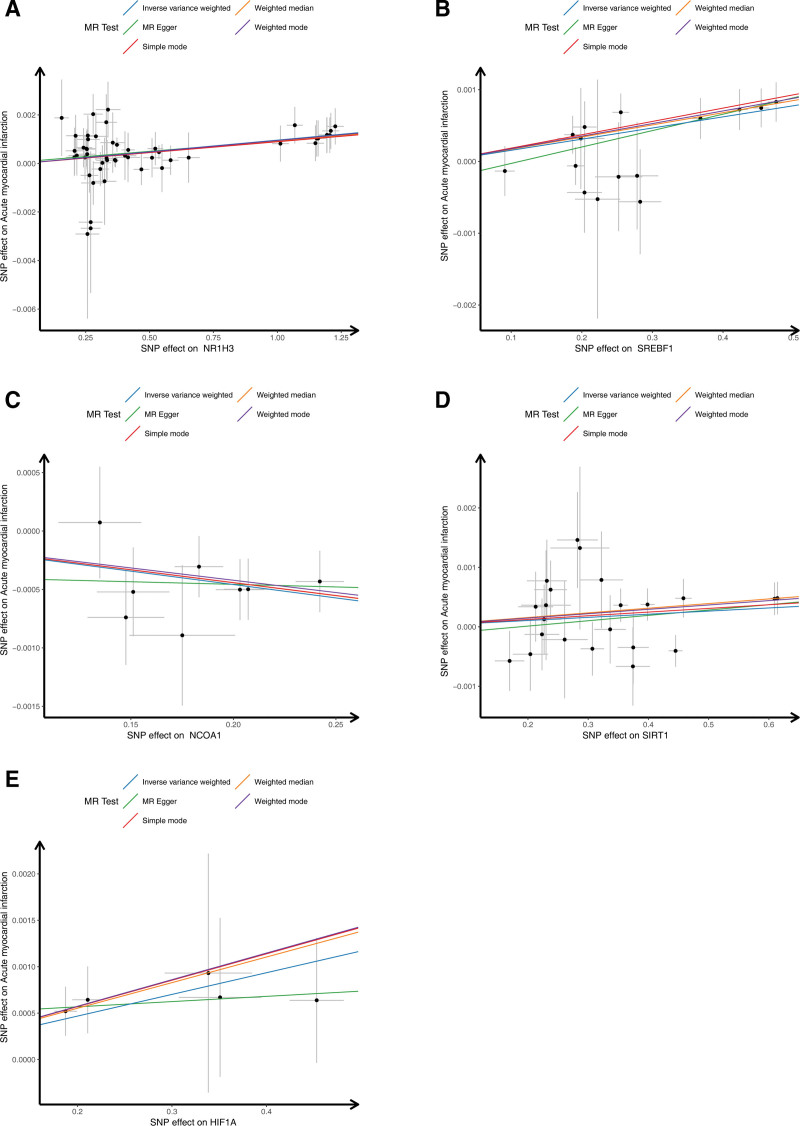
The correlation of 5 circadian rhythm-related hub genes with AMI. AMI = acute myocardial infarction, MR = Mendelian randomization, SNP = single nucleotide polymorphism.

**Figure 2.**
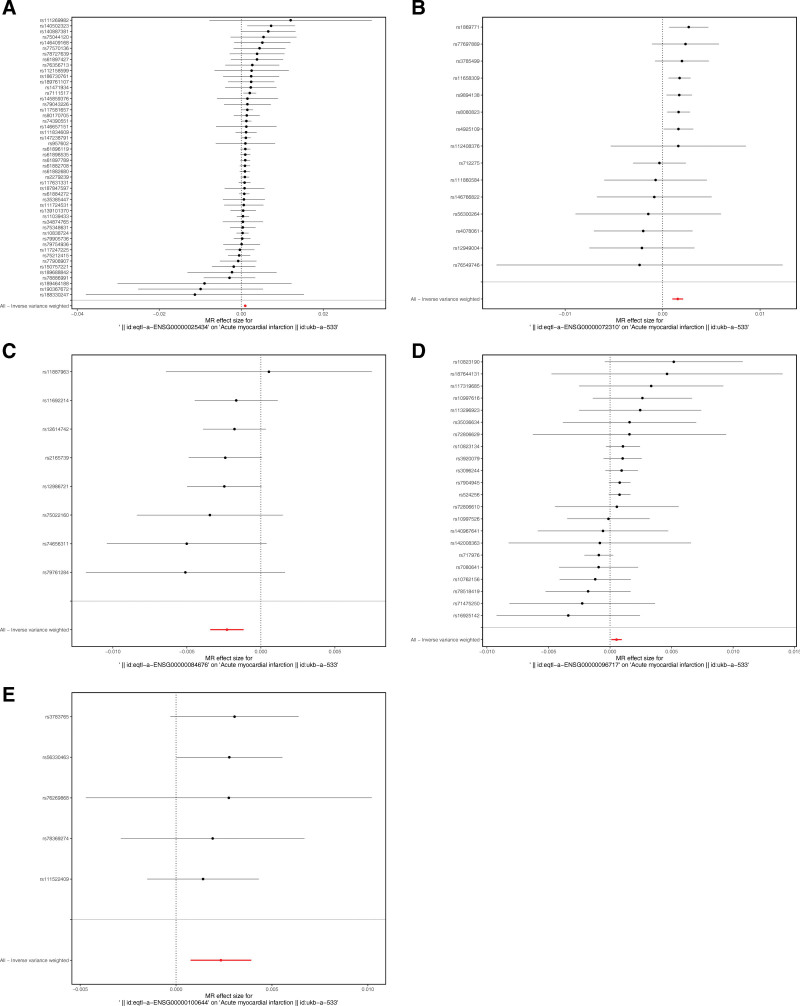
The risk of 5 circadian rhythm-related hub genes with AMI. AMI = acute myocardial infarction, MR = Mendelian randomization.

In this study, the CRRGs were exposure factors and the AMI was an outcome variable. By filtering, a total of 222 independent SNPs were selected as IVs (Table S1, Supplemental Digital Content, https://links.lww.com/MD/R153). Importantly, there were 5 significant exposure factors for AMI (Table 1). Among them, NR1H3 (IVW: OR = 1.0009, 95% CI = 1.0005–1.0013, *P* < .0001), SREBF1 (IVW: OR = 1.0015, 95% CI = 1.0007–1.0022, *P* = .0003), SIRT1 (IVW: OR = 1.0007, 95% CI = 1.0001–1.0013, *P* = .0184), and HIF1A (IVW: OR = 1.0022, 95% CI = 1.0004–1.0039, *P* = .0140) were risk factors for AMI patients, while NCOA1 (IVW: OR = 0.9984, 95% CI = 0.9969–0.9998, *P* = .0230) was a protective factor for AMI patients. Notably, after false discovery rate correction, the corrected *P*-values (fdr_p) of all genes were <.05. This indicates that under the premise of controlling the false positive rate, the associations between these genes and AMI are statistically significant, which ensures the reliability of the results (Table S2, Supplemental Digital Content, https://links.lww.com/MD/R153). Likewise, the scatter diagrams and the forest plots of SNPs-genes effect and SNPs-AMI effect demonstrated a high extent of conformity between the study results and the IVW results (Figs. 1 and 2). Moreover, the IVs spots were evenly distributed around the IVW lines, which proved that the TSMR results met Mendel second law of random grouping (Fig. S1, Supplemental Digital Content, https://links.lww.com/MD/R153).

**Table 1:** Five significant exposure factors for AMI.

Symbol	Outcome	Exposure	Method	NSNP	B	SE	*P*-val	OR	or_lci95	or_uci95
NR1H3	Acute myocardial infarction ‖ id:ukb-a-533	‖ id:eqtl-a-ENSG00000025434	Inverse variance weighted	32	0.000897237	0.000218024	3.87E−05	1.000897639	1.000470021	1.001325441
SREBF1	Acute myocardial infarction ‖ id:ukb-a-533	‖ id:eqtl-a-ENSG00000072310	Inverse variance weighted	9	0.001459036	0.000399865	.000263443	1.001460101	1.000675529	1.002245288
NCOA1	Acute myocardial infarction ‖ id:ukb-a-533	‖ id:eqtl-a-ENSG00000084676	Inverse variance weighted	5	-0.00165047	0.000725946	.02299341	0.998350891	0.996931393	0.99977241
SIRT1	Acute myocardial infarction ‖ id:ukb-a-533	‖ id:eqtl-a-ENSG00000096717	Inverse variance weighted	12	0.00073127	0.000310111	.018369086	1.000731537	1.00012346	1.001339983
HIF1A	Acute myocardial infarction ‖ id:ukb-a-533	‖ id:eqtl-a-ENSG00000100644	Inverse variance weighted	4	0.002184784	0.000889368	.014027504	1.002187172	1.00044172	1.003935669

AMI = acute myocardial infarction.

### 3.2. Hub genes affected AMI only in forward direction

**Figure 3.**
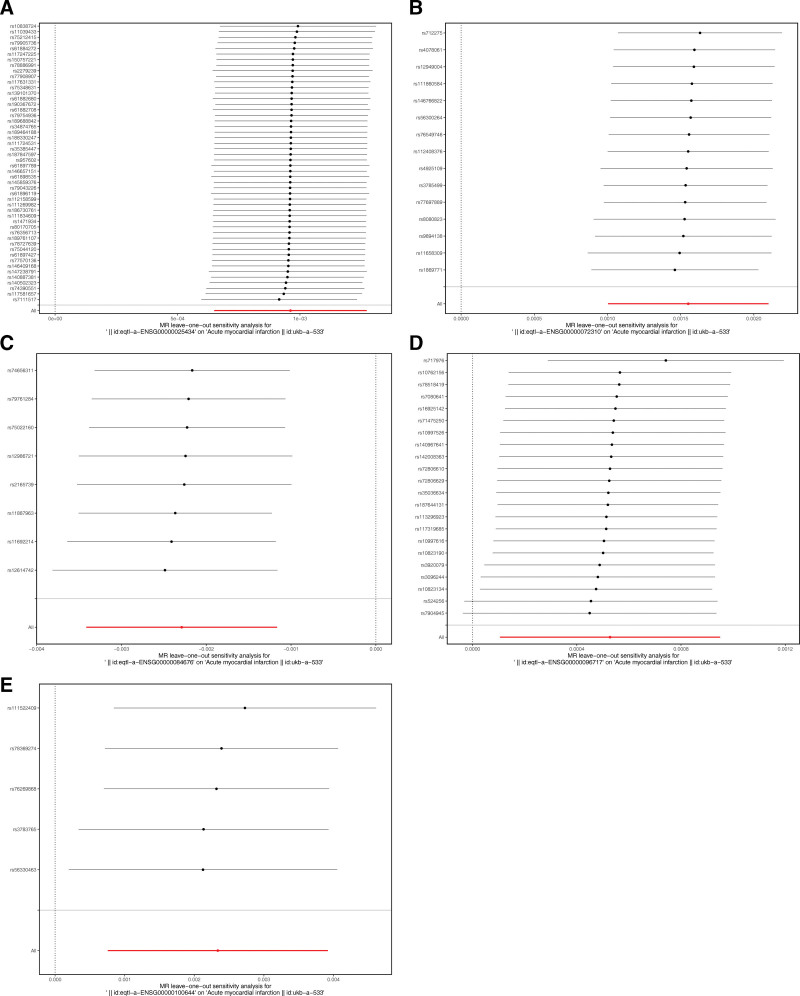
The LOO analyses the TSMR results of 5 hub genes sffected AMI. AMI = acute myocardial infarction, LOO = leave-one-out, MR = Mendelian randomization, TSMR = two-sample Mendelian randomization.

To verify the above TSMR results, sensitivity analysis was performed. There was no heterogeneity between the hub genes and AMI (*Q* > 0.05). Meanwhile, no confounding factors had a significant effect on TSMR results (Table 2). Moreover, the LOO analysis indicated that the TSMR results were stable and reliable (Fig. 3). Finally, The results of the MR-Steiger test indicated that for the hub genes, the Steiger-direction outcomes were positive (TRUE, *P* < .05) (Table 3).

**Table 2:** The TSMR result of 5 exposure factors for AMI.

Outcome	Exposure	Gene	Heterogeneity	pleiotropy	Outlier examination by MR-PRESSO
Acute myocardial infarction ‖ id:ukb-a-533	eqtl-a-ENSG00000025434	NR1H3	0.9901	0.7063	0.993
Acute myocardial infarction ‖ id:ukb-a-533	eqtl-a-ENSG00000072310	SREBF1	0.7975	0.2978	0.838
Acute myocardial infarction ‖ id:ukb-a-533	eqtl-a-ENSG00000084676	NCOA1	0.8911	0.6132	0.927
Acute myocardial infarction ‖ id:ukb-a-533	eqtl-a-ENSG00000096717	SIRT1	0.4737	0.5712	0.475
Acute myocardial infarction ‖ id:ukb-a-533	eqtl-a-ENSG00000100644	HIF1A	0.9505	0.5001	0.936

AMI = acute myocardial infarction, TSMR = two-sample Mendelian randomization.

**Table 3:** The effect of hub genes on AMI occurrence.

Exposure	Outcome	Snp_r2.exposure	Snp_r2.outcome	Correct_causal_direction	Steiger_*P*
NR1H3	Acute myocardial infarction	0.6396	0.0002	TRUE	0
SREBF1	Acute myocardial infarction	0.1713	0.0001	TRUE	0
NCOA1	Acute myocardial infarction	0.0485	<0.0001	TRUE	2.38 × 10^−285^
SIRT1	Acute myocardial infarction	0.4891	<0.0001	TRUE	0
HIF1A	Acute myocardial infarction	0.0256	<0.0001	TRUE	1.55 × 10^−143^

AMI = acute myocardial infarction.

### 3.3. Hub genes were enriched in 808 GO items and 31 KEGG pathways

**Figure 4.**
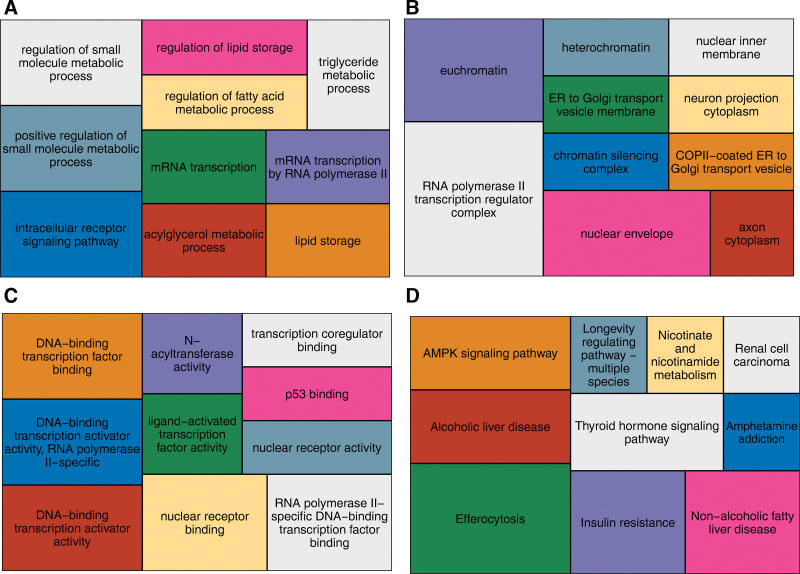
Hub genes in GO items and KEGG pathways. (A) Biological process; (B) cellular component; (C) molecular function; and (D) KEGG pathways. GO = gene ontology, KEGG = Kyoto Encyclopedia of Genes and Genomes.

Here, we further explored the function of genes based on the GO and KEGG enrichment analyses. The hub genes were significantly enriched in 808 GO items, comprising 752 BPs (e.g. positive regulation of small molecule metabolic process, intracellular receptor signaling pathway, regulation of small molecule metabolic process, etc) (Fig. 4A, Table S3, Supplemental Digital Content, https://links.lww.com/MD/R153), 13 CCs (e.g. RNA polymerase II transcription regulator complex, euchromatin, nuclear envelope, etc) (Fig. 4B, Table S4, Supplemental Digital Content, https://links.lww.com/MD/R153), and 43 MFs (e.g. nuclear receptor binding, RNA polymerase II-specific DNA-binding TF binding, DNA-binding transcription activator activity, etc) items (*P* < .05) (Fig. 4C, Table S5, Supplemental Digital Content, https://links.lww.com/MD/R153). Furthermore, the hub genes were significantly enriched in 31 KEGG pathways (e.g. efferocytosis, insulin resistance, AMPK signaling pathway, etc) (Fig. 4D, Table S6, Supplemental Digital Content, https://links.lww.com/MD/R153). The results suggested that hub genes might influence nuclear envelope, nuclear receptor binding, AMPK signaling pathway, and efferocytosis in AMI development.

### 3.4. The localization and expression of hub genes were explored

**Figure 5.**
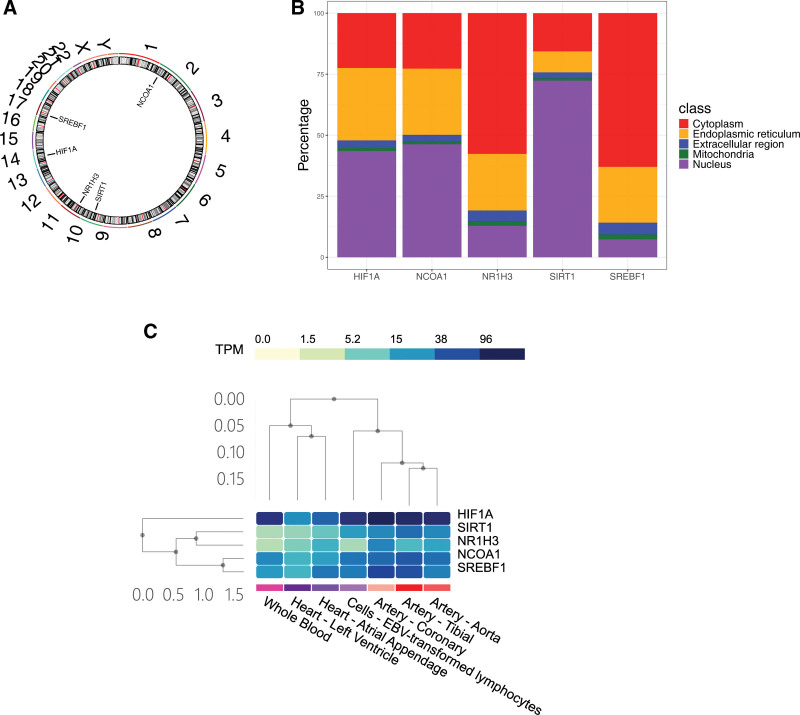
The localization and expression of hub genes. (A) Chromosome localization of 5 hub genes; (B) The percentage of 5 hub genes in cells localization; (C) The expression of 5 hub genes in tissues.

After obtaining the hub genes, we conducted further studies around these genes. Chromosome localization results showed that NR1H3, SREBF1, SIRT1, HIF1A, and NCOA1 were located on chromosomes 11, 17, 10, 14, and 2, respectively (Fig. 5A). Particularly, the NR1H3 and SREBF1 were mainly located in the cytoplasm, while the other 3 hub genes were mainly located in the nucleus (Fig. 5B). Afterwards, the expression levels of HIF1A, NCOA1, and SREBF1 were predicted to be the highest in neutrophils (Fig. S2A–C, Supplemental Digital Content, https://links.lww.com/MD/R153). Also, the expression level of NR1H3 was predicted to be the highest in intermediate monocytes (Fig. S2D, Supplemental Digital Content, https://links.lww.com/MD/R153), and the expression level of SIRT1 was predicted to be the highest in naïve CD4 T cells (Fig. S2E, Supplemental Digital Content, https://links.lww.com/MD/R153). Interestingly, the expression levels of HIF1A and SREBF1 were higher in the heart (left ventricle and atrial appendage) and artery (aorta, tibial, and coronary) (Fig. 5C).

### 3.5. Two molecular networks were constructed based on the hub genes

**Figure 6.**
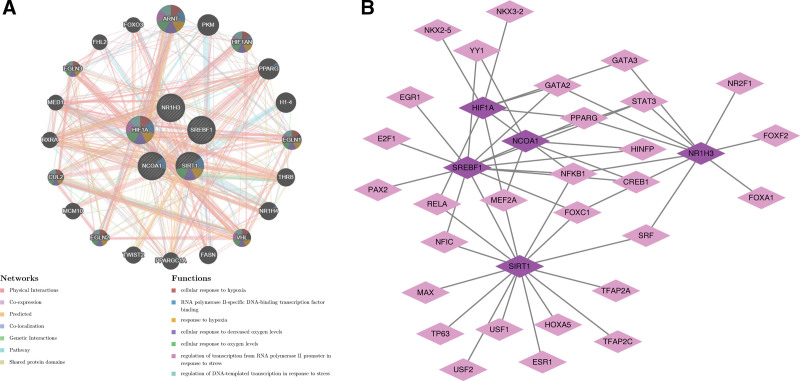
Two molecular networks. (A) Five hub genes and the top 20 genes constructed a GGI network; (B) 29 TFs associated with hub genes framed TF-mRNA network. GGI = gene–gene interaction, TF = transcription factor.

A GGI network was constructed based on the 5 hub genes and the top 20 genes, including HIF1A-ARNT, NCOA1-PPARG, NR1H3-HIF1AN, etc (Fig. 6A). More specially, the above 25 genes were mainly co-enriched in the cellular response to hypoxia. Then, a sum of 29 TFs associated with hub genes were predicted, and then a TF-mRNA network was generated, involving 34 nodes and 50 edges (Fig. 6B, Table S7, Supplemental Digital Content, https://links.lww.com/MD/R153). Notably, NFKB1 was involved in the regulation of NR1H3, SREBF1, SIRT1, and HIF1A.

## 4. Discussion

AMI is a life-threatening form of CHD, with morbidity rates rising rapidly.^[27]^ Without timely and effective treatment, AMI remains a highly lethal cardiovascular condition, making early detection and prevention essential. In this study, we conducted a MR analysis to investigate the relationship between CRRGs and AMI. Our analysis identified 5 hub genes influencing AMI: NR1H3, SREBF1, SIRT1, and HIF1A were found to be risk factors, while NCOA1 was identified as a protective factor. The genes influence the development of AMI through their tissue-specific regulation of lipid metabolism, inflammatory responses, and energy homeostasis. Circadian rhythm genes integrate these pathways, establishing temporally coordinated protective mechanisms within the cardiovascular system.^[28]^ Disruption of these rhythms induces metabolic dysregulation, inflammatory bursts, and autonomic nervous system imbalance, significantly increasing susceptibility to AMI.

This study emphasized the association between 5 key exposure factors and AMI, with NR1H3, SREBF1, SIRT1, and HIF1A identified as risk factors, while NCOA1 served as a protective factor. FXR, encoded by NR1H3, is a nuclear receptor primarily expressed in the liver and intestines, where it regulates bile acid, lipid, and glucose metabolism.^[29]^ By modulating genes involved in lipid metabolism, FXR reduces lipid deposition and the risk of atherosclerosis, which may indirectly influence the occurrence of AMI. Its anti-inflammatory properties also help reduce myocardial injury by inhibiting inflammatory factor expression.^[30]^ Previous research on this topic is limited, though existing studies align with our findings. Additionally, Tingting Li et al found that SREBF1 regulates lipid metabolism, influencing the development of cardiovascular diseases.^[31]^ It encodes a key TF involved in the synthesis of cholesterol, fatty acids and triglycerides. The hyperactivity of SREBF1 leads to lipid accumulation and atherosclerosis, increasing AMI risk.^[31]^ It is also closely linked to metabolic syndrome, a major risk factor for AMI.^[32]^ Chunjuan Chen showed that the SIRT1/NF-κB/sCD40L axis is elevated in AMI patients, with SIRT1 levels positively correlating with cardiac troponin T.^[33]^ SIRT1, a NAD^+^ dependent deacetylase, regulates cellular metabolism, aging, and stress responses. It affects the progression of AMI by regulating energy metabolism, oxidative stress and inflammatory response.^[34]^ Additionally, During the process of myocardial ischemia and hypoxia, overactivation of SIRT1 inhibits angiogenesis, that affects myocardial blood supply and promots myocardial necrosis. SIRT1 also impacts insulin sensitivity, fat metabolism, and oxidative stress, which affects myocardial cell damage.^[35,36]^ These processes collectively promote the initiation and progression of atherosclerosis, and directly lead to myocardial necrosis, thus triggering AMI. Ratul Datta Chaudhuri discovered novel crosstalk between HIF1A and NFκB pathways, which influences cardiomyocyte apoptosis by regulating bnip3 and ho-1 expression during myocardial infarction.^[37]^ Moreover HIF1A, activated under hypoxic conditions, regulates the expression of genes related to the hypoxic response. It promotes increased vascular permeability via VEGF, accelerating lipid infiltration and intimal hyperplasia. Concurrently, it exacerbates inflammatory responses, driving the pathological progression of atherosclerosis. Under chronic hypoxia, HIF1A upregulates BNIP3/NIX expression, triggering excessive mitophagy and ultimately accelerating myocardial fibrosis and apoptosis, thereby inducing myocardial injury following AMI.^[38,39]^ In addition NCOA1 is a transcriptional coactivator that enhances nuclear receptor-mediated gene transcription. It plays a role in regulating lipid and glucose metabolism, affecting the risk of atherosclerosis and cardiovascular diseases.^[40,41]^ It may also promote anti-inflammatory responses and improve metabolic states, exerting a protective effect on the cardiovascular system and potentially reducing the risk of AMI. These studies suggest a causal relationship between NR1H3, SREBF1, SIRT1, HIF1A, NCOA1, and AMI, supporting our results. However, further research is required to strengthen the evidence and confirm these findings.

In combination with the GGI network, the top gene identified is aryl hydrocarbon receptor nuclear translocator (ARNT), which plays a pivotal role in AMI due to its function as a TF. ARNT acts as a cofactor for several important TFs, including HIF1A and aryl hydrocarbon receptor (AHR). Its mechanism in AMI primarily involves regulating the hypoxic response, oxidative stress, and inflammation through interactions with HIF1A and AHR.^[42–44]^ While ARNT activation may protect cardiomyocytes in some cases, excessive activity can worsen pathological damage. Additionally, TF-mRNA regulatory network suggests that NFKB1 co-regulates 4 hub genes. It plays a crucial role in AMI pathology by influencing inflammation, oxidative stress, and apoptosis.^[45]^ Moderate activation of the NFKB1 signaling pathway can be protective for cardiomyocyte survival, but excessive activation may exacerbate myocardial injury and accelerate the progression of AMI.^[46,47]^

Innate immune cells play a critical role in tissue damage and repair following AMI. In this study, HIF1A and SIRT1 are highly expressed in intermediate monocytes and naive CD4^+^ T cells. Intermediate monocytes are a subset of circulating monocytes, characterized by high CD14 and low CD16 expression (CD14^++^CD16^+^).^[48]^ Due to their pro-inflammatory nature and the production of large amounts of cytokines such as tumor necrosis factor-α (TNF-α) and interleukin-1β (IL-1β), they play a significant role in AMI. These monocytes are involved in the early inflammatory response following AMI, where excessive or prolonged inflammation can worsen myocardial injury and hinder the healing process. Furthermore, they contribute to myocardial remodeling, a critical aspect of tissue repair after ischemic heart injury. This process is often associated with fibrosis and adverse remodeling, which can eventually lead to heart failure.^[49,50]^ The development, distribution, quantity, and functional activity of monocytes are closely linked to circadian rhythms. On one hand, the secretion efficiency of cytokines (TNF-α, IL-1β) by monocytes exhibits significant rhythmicity, peaking in the early morning. This nocturnal peak is primarily attributed to the heightened responsiveness of monocytes.^[51]^ Notably, this time window coincides with the high incidence period of AMI.^[52]^ Disruptions in circadian rhythms caused by factors such as staying up late or jet lag can disturb the rhythmic fluctuations in monocyte numbers and functional balance.^[53]^ This may lead to abnormal recruitment of monocytes to injury sites and excessive enhancement of their pro-inflammatory activity, thereby exacerbating myocardial damage in inflammation-related diseases such as AMI, impairing tissue repair, and even increasing the risk of heart failure. Moreover, CD4^+^ T cells, particularly naive CD4^+^ T cells, also play a pivotal role in immune regulation. Following ischemic damage to the myocardium, injured tissue releases danger signals that recruit immune cells to the site. Naive CD4^+^ T cells, with the assistance of antigen-presenting cells like dendritic cells, become activated and differentiate into various effector T cell subsets. These subsets regulate the intensity and duration of the inflammatory response during AMI. Each type of effector T cell plays a specific role in the inflammation, fibrosis, and repair processes, and an excessive or inappropriate T cell response can further exacerbate myocardial damage.^[54,55]^ Furthermore, studies have indicated that chronic inflammation exerts a lasting impact on the circadian rhythms of peripheral immune cells, and such circadian abnormalities, in turn, affect the functional regulation of CD4^+^ T cells.^[56]^ Circadian rhythm disruption also suppresses BMAL1 expression, leading to aberrant differentiation of CD4^+^ T cells into Th1/Th17 cells, excessive secretion of pro-inflammatory cytokines, and functional impairment of Treg cells. Additionally, it inhibits their ability to assist macrophages in clearing necrotic tissue.^[57]^ These effects may exacerbate myocardial injury and hinder repair processes in AMI, thereby increasing the risk of heart failure. This underscores that maintaining normal circadian rhythms or targeted modulation of the rhythmic functions of monocytes and CD4^+^ T cells could potentially optimize immunotherapy for AMI and improve patient prognosis. The high expression levels of HIF1A and SIRT1 in intermediate monocytes and naive CD4^+^ T cells highlight the crucial roles these genes play in orchestrating immune responses, tissue damage control, and repair following AMI.

This study, utilizing data from online databases and the TSMR method, identified 4 risk factors (NR1H3, SREBF1, SIRT1, and HIF1A) and one protective factor (NCOA1) that may directly influence the occurrence of AMI. These findings provide a theoretical foundation for the diagnosis, treatment, and exploration of the pathogenic mechanisms of AMI, with a particular emphasis on hub genes.The cell/tissue-specific expression profiles of these genes (e.g., high expression of HIF1A and SREBF1 in cardiac and arterial tissues, and high expression of SIRT1 in intermediate monocytes and naïve CD4^+^ T cells) could serve as potential biomarkers for AMI. Monitoring their expression levels may aid in the early identification of high-risk populations and assessment of disease severity. At the therapeutic level, these genes represent promising intervention targets. For instance, optimizing the timing of statin administration based on the lipid metabolism pathways regulated by NR1H3 (aligning with its circadian rhythm-related functions) could enhance treatment efficacy. Meanwhile, the anti-inflammatory and metabolic regulatory pathways associated with NCOA1 offer new directions for developing protective drugs against AMI. In terms of prevention, given the close relationship between core gene functions and circadian rhythms, maintaining a normal circadian rhythm (e.g., avoiding sleep deprivation and jet lag) is crucial for regulating gene expression and reducing the early morning peak incidence of AMI. This is particularly relevant for cardiovascular health management in populations prone to rhythm disruptions, such as shift workers, providing practical significance for risk mitigation.

However, this study has several limitations. First, experimental validation remains insufficient, and the conclusions heavily rely on bioinformatics analysis and computational predictions, lacking direct support from wet-lab experiments (e.g., functional cellular assays and molecular mechanism verification). Second, the effect sizes of the core genes on AMI are relatively small, which may be attributed to gene-environment interactions or polygenic synergistic effects. Third, the identified core genes are primarily involved in lipid metabolism and vascular inflammation-related pathways, linking them to AMI through atherosclerosis, while other disease mechanisms remain unexplored. Fourth, the screening of core genes depended on retrospective GWAS data from European populations, which inherently carry potential confounding factors. Moreover, genetic polymorphisms in European populations differ from those in Chinese populations, potentially limiting the generalizability of our findings to Chinese cohorts. In future work, we will further investigate the specific functions of prognosis-related genes in the pathogenesis and progression of AMI to clarify their potential as diagnostic biomarkers or therapeutic targets. Additionally, we plan to employ cellular experiments and establish AMI animal models (e.g., myocardial ischemia-reperfusion models in rats) to elucidate the molecular mechanisms through which core genes regulate AMI. Concurrently, we will advance in vitro screening and in vivo validation of targeted drugs. Furthermore, we will prioritize the design of prospective cohort studies in Chinese populations, incorporating region-specific characteristics to systematically validate the association between core genes and AMI, thereby enhancing the cross-population applicability and clinical translational value of our findings.

## 5. Conclusion

Overall, this study supports a causal relationship between circadian rhythm genes and AMI, offering new insights into the treatment and prevention of AMI patients.

## Author contributions

**Conceptualization:** Man-Hua Chen.

**Data curation:** Song Peng, Yan Leng.

**Formal analysis:** Song Peng, Yan Leng.

**Funding acquisition:** Song Peng.

**Methodology:** Song Peng, Yan Leng.

**Software:** Song Peng, Yan Leng.

**Software, Methodology, Data curation, Formal analysis:** Song Peng, Yan Leng.

**Writing – review & editing:** Song Peng, Man-Hua Chen.

## Supplementary Material


