# Supplementary material for: Discovery of circadian rhythm-related hub genes in acute myocardial infarction: A two-sample Mendelian randomization study
Source: Medicine (Baltimore). 2026 Jan 16;105(3):e47228. doi: 10.1097/MD.0000000000047228 (PMC12826203; doi:10.1097/MD.0000000000047228)

Table S1 222 independent SNPs were selected as IVs

outcome	exposure	method	nsnp	b	se	pval	lo_ci	up_ci	or	or_lci95	or_uci95
Acute myocardial infarction || id:ukb-a-533	|| id:eqtl-a-ENSG00000007171	MR Egger	3	-0.004416999	0.004769374	0.524407793	-0.013764972	0.004930974	0.995592742	0.986329332	1.004943151
Acute myocardial infarction || id:ukb-a-533	|| id:eqtl-a-ENSG00000007171	Weighted median	3	-0.001220073	0.001270246	0.336542815	-0.003710412	0.001268952	0.998780015	0.996296463	1.001269757
Acute myocardial infarction || id:ukb-a-533	|| id:eqtl-a-ENSG00000007171	Inverse variance weighted	3	-0.001264458	0.00120344	0.29339506	-0.003623201	0.001094285	0.998736341	0.996383355	1.001094884
Acute myocardial infarction || id:ukb-a-533	|| id:eqtl-a-ENSG00000007171	Simple mode	3	0.00102609	0.00218289	0.684505916	-0.003251196	0.005303375	1.001026616	0.996754084	1.005317463
Acute myocardial infarction || id:ukb-a-533	|| id:eqtl-a-ENSG00000007171	Weighted mode	3	-0.001771779	0.00124588	0.290759817	-0.004212543	0.000668986	0.99822979	0.995796317	1.00066921
Acute myocardial infarction || id:ukb-a-533	|| id:eqtl-a-ENSG00000008405	MR Egger	3	0.000397449	0.004362947	0.94216589	-0.008153927	0.008948824	1.000397528	0.991879226	1.008988985
Acute myocardial infarction || id:ukb-a-533	|| id:eqtl-a-ENSG00000008405	Weighted median	3	-0.000125392	0.001323836	0.924538118	-0.002720111	0.002469326	0.999874616	0.997283586	1.002472377
Acute myocardial infarction || id:ukb-a-533	|| id:eqtl-a-ENSG00000008405	Inverse variance weighted	3	1.85E-05	0.001248917	0.988197195	-0.002429403	0.002466354	1.000018476	0.997573546	1.002469398
Acute myocardial infarction || id:ukb-a-533	|| id:eqtl-a-ENSG00000008405	Simple mode	3	-1.89E-05	0.001973075	0.993235531	-0.003886103	0.003848352	0.999981125	0.996121438	1.003855766
Acute myocardial infarction || id:ukb-a-533	|| id:eqtl-a-ENSG00000008405	Weighted mode	3	-0.000177147	0.001435208	0.913052839	-0.002990155	0.002635861	0.999822869	0.997014311	1.002639338

Acute myocardial infarction || id:ukb-a-533	|| id:eqtl-a-ENSG00000013583	MR Egger	3	-0.000097315	0.001955913	0.706085656	-0.004806739	0.00286044	0.999027324	0.995204795	1.002864535
Acute myocardial infarction || id:ukb-a-533	|| id:eqtl-a-ENSG00000013583	Weighted median	3	-0.000285265	0.00070682	0.686514972	-0.001670632	0.00110010 2	0.999714776	0.998330763	1.001100707
Acute myocardial infarction || id:ukb-a-533	|| id:eqtl-a-ENSG00000013583	Inverse variance weighted	3	-0.000254037	0.00068073 3	0.709013171	-0.001588274	0.0010802	0.999745995	0.998412986	1.001080783
Acute myocardial infarction || id:ukb-a-533	|| id:eqtl-a-ENSG00000013583	Simple mode	3	-0.000402167	0.00108379 5	0.746202876	-0.002526405	0.00172207 1	0.999597914	0.997476783	1.001723554
Acute myocardial infarction || id:ukb-a-533	|| id:eqtl-a-ENSG00000013583	Weighted mode	3	-0.000340477	0.00079967 3	0.711716276	-0.001907836	0.00122688 1	0.999659581	0.998093983	1.001227634
Acute myocardial infarction || id:ukb-a-533	|| id:eqtl-a-ENSG00000025434	MR Egger	32	0.000607915	0.00044900 8	0.185878117	-0.000027214	0.00148797	1.0006081	0.999727897	1.001489078
Acute myocardial infarction || id:ukb-a-533	|| id:eqtl-a-ENSG00000025434	Weighted median	32	0.000777758	0.00028469 1	0.006296252	0.000219764	0.00133575 1	1.00077806	1.000219788	1.001336644
Acute myocardial infarction || id:ukb-a-533	|| id:eqtl-a-ENSG00000025434	Inverse variance weighted	32	0.000897237	0.00021802 4	3.87E-05	0.000469911	0.00132456 3	1.000897639	1.000470021	1.001325441
Acute myocardial infarction || id:ukb-a-533	|| id:eqtl-a-ENSG00000025434	Simple mode	32	0.000754422	0.00033516 6	0.031627324	9.75E-05	0.00141134 7	1.000754706	1.000097501	1.001412343
Acute myocardial infarction || id:ukb-a-533	|| id:eqtl-a-ENSG00000025434	Weighted mode	32	0.000806756	0.00025579 3	0.003566125	0.000305402	0.00130811 1	1.000807082	1.000305449	1.001308967
Acute myocardial infarction || id:ukb-a-533	|| id:eqtl-a-ENSG00000026103	MR Egger	10	-0.000628821	0.00099217 2	0.543913919	-0.002573478	0.00131583 6	0.999371377	0.99742983	1.001316702

Acute myocardial infarction || id:ukb-a-533	|| id:eqtl-a-ENSG00000026103	Weighted median	10	0.000445366	0.000556361	0.423421488	-0.0006451	0.001535833	1.000445466	0.999355108	1.001537013
Acute myocardial infarction || id:ukb-a-533	|| id:eqtl-a-ENSG00000026103	Inverse variance weighted	10	0.000519279	0.000514295	0.312643038	-0.000488738	0.001527296	1.000519414	0.999511381	1.001528463
Acute myocardial infarction || id:ukb-a-533	|| id:eqtl-a-ENSG00000026103	Simple mode	10	0.000433793	0.00153251	0.783533286	-0.002569926	0.003437512	1.000433887	0.997433374	1.003443427
Acute myocardial infarction || id:ukb-a-533	|| id:eqtl-a-ENSG00000026103	Weighted mode	10	0.00039492	0.000581562	0.514177442	-0.000744942	0.001534781	1.000394998	0.999255336	1.001535959
Acute myocardial infarction || id:ukb-a-533	|| id:eqtl-a-ENSG00000043591	MR Egger	3	0.001082234	0.002342781	0.724507845	-0.003509617	0.005674085	1.001108282	0.996496534	1.005690213
Acute myocardial infarction || id:ukb-a-533	|| id:eqtl-a-ENSG00000043591	Weighted median	3	0.00138487	0.000808734	0.086824445	-0.000200249	0.002969989	1.00138583	0.999799771	1.002974404
Acute myocardial infarction || id:ukb-a-533	|| id:eqtl-a-ENSG00000043591	Inverse variance weighted	3	0.001434182	0.000763558	0.060341823	-6.24E-05	0.002930756	1.001435211	0.99993761	1.002935055
Acute myocardial infarction || id:ukb-a-533	|| id:eqtl-a-ENSG00000043591	Simple mode	3	0.001217021	0.001032528	0.35976125	-0.000806734	0.003240775	1.001217761	0.999193592	1.003246032
Acute myocardial infarction || id:ukb-a-533	|| id:eqtl-a-ENSG00000043591	Weighted mode	3	0.001395438	0.000845481	0.240636154	-0.000261705	0.00305258	1.001396412	0.999738329	1.003057244
Acute myocardial infarction || id:ukb-a-533	|| id:eqtl-a-ENSG00000049246	MR Egger	8	-0.001083939	0.001232428	0.4129571	-0.003499499	0.00133162	0.998916648	0.996506617	1.001332507
Acute myocardial infarction || id:ukb-a-533	|| id:eqtl-a-ENSG00000049246	Weighted median	8	9.72E-06	0.000835641	0.990721226	-0.001628139	0.001647575	1.000009718	0.998373186	1.001648933

Acute myocardial infarction || id:ukb-a-533	|| id:eqtl-a-ENSG00000049246	Inverse variance weighted	8	0.000595097	0.000798608	0.456169704	-0.000970173	0.002160368	1.000595274	0.999030297	1.002162703
Acute myocardial infarction || id:ukb-a-533	|| id:eqtl-a-ENSG00000049246	Simple mode	8	-0.000115345	0.001496963	0.940738018	-0.003049393	0.002818704	0.999884662	0.996955252	1.00282268
Acute myocardial infarction || id:ukb-a-533	|| id:eqtl-a-ENSG00000049246	Weighted mode	8	-0.000768228	0.000873756	0.408445697	-0.002480789	0.000944334	0.999232068	0.997522286	1.00094478
Acute myocardial infarction || id:ukb-a-533	|| id:eqtl-a-ENSG00000049247	MR Egger	5	0.001188663	0.001388657	0.454932559	-0.001533104	0.003910443	1.00118937	0.998468071	1.003918086
Acute myocardial infarction || id:ukb-a-533	|| id:eqtl-a-ENSG00000049247	Weighted median	5	0.00012988	0.000777344	0.867386263	-0.001393794	0.00165339 5	1.000129809	0.998607177	1.001654763
Acute myocardial infarction || id:ukb-a-533	|| id:eqtl-a-ENSG00000049247	Inverse variance weighted	5	5.32E-05	0.001138688	0.962770522	-0.002178677	0.002284979	1.000053152	0.997823694	1.002287591
Acute myocardial infarction || id:ukb-a-533	|| id:eqtl-a-ENSG00000049247	Simple mode	5	-0.000322913	0.001347929	0.822444378	-0.002964854	0.002319027	0.999677139	0.997039537	1.002321718
Acute myocardial infarction || id:ukb-a-533	|| id:eqtl-a-ENSG00000049247	Weighted mode	5	0.000233964	0.000819495	0.789436235	-0.001372246	0.001840175	1.000233992	0.998628695	1.001841869
Acute myocardial infarction || id:ukb-a-533	|| id:eqtl-a-ENSG00000050748	MR Egger	3	2.63E-05	0.001122857	0.98509593	-0.002174508	0.002227092	1.000026293	0.997827855	1.002229574
Acute myocardial infarction || id:ukb-a-533	|| id:eqtl-a-ENSG00000050748	Weighted median	3	-0.000676564	0.00069387	0.329531319	-0.002036548	0.000683421	0.999323665	0.997965524	1.000683655
Acute myocardial infarction || id:ukb-a-533	|| id:eqtl-a-ENSG00000050748	Inverse variance weighted	3	-0.000884751	0.000659637	0.179833004	-0.002177639	0.000408138	0.999115641	0.99782473	1.000408221

Acute myocardial infarction || id:ukb-a-533	|| id:eqtl-a-ENSG00000050748	Simple mode	3	-0.000734716	0.001350055	0.640857667	-0.003380824	0.001911391	0.999265553	0.996624885	1.001913219
Acute myocardial infarction || id:ukb-a-533	|| id:eqtl-a-ENSG00000050748	Weighted mode	3	-0.000664595	0.00072628 3	0.456755314	-0.002088109	0.000758919	0.999335626	0.99791407	1.000759207
Acute myocardial infarction || id:ukb-a-533	|| id:eqtl-a-ENSG00000054118	MR Egger	5	-0.004660376	0.00289673 5	0.206022996	-0.010337977	0.001017225	0.995350467	0.989715276	1.001017742
Acute myocardial infarction || id:ukb-a-533	|| id:eqtl-a-ENSG00000054118	Weighted median	5	0.000225925	0.00153932 7	0.883314572	-0.002791156	0.003243007	1.000225951	0.997212735	1.003248271
Acute myocardial infarction || id:ukb-a-533	|| id:eqtl-a-ENSG00000054118	Inverse variance weighted	5	0.001985205	0.00160712 3	0.216736128	-0.001164757	0.005135166	1.001987177	0.998835921	1.005148374
Acute myocardial infarction || id:ukb-a-533	|| id:eqtl-a-ENSG00000054118	Simple mode	5	0.004671215	0.00305376 7	0.200840029	-0.001314169	0.010656599	1.004682142	0.998686694	1.010713583
Acute myocardial infarction || id:ukb-a-533	|| id:eqtl-a-ENSG00000054118	Weighted mode	5	-0.000139735	0.00147309 9	0.928989402	-0.003027009	0.002747538	0.999860274	0.996977568	1.002751316
Acute myocardial infarction || id:ukb-a-533	|| id:eqtl-a-ENSG00000054277	MR Egger	4	-0.001588073	0.00114291 7	0.299155481	-0.003828191	0.000652044	0.998413187	0.996179127	1.000652257
Acute myocardial infarction || id:ukb-a-533	|| id:eqtl-a-ENSG00000054277	Weighted median	4	-0.000590373	0.00057684 7	0.306095934	-0.001720993	0.000540247	0.999409801	0.998280487	1.000540393
Acute myocardial infarction || id:ukb-a-533	|| id:eqtl-a-ENSG00000054277	Inverse variance weighted	4	-0.000341442	0.00083133 9	0.681282794	-0.001970866	0.001287982	0.999658616	0.998031074	1.001288812
Acute myocardial infarction || id:ukb-a-533	|| id:eqtl-a-ENSG00000054277	Simple mode	4	-0.000448866	0.00135857 5	0.762830522	-0.003111673	0.002213942	0.999551235	0.996893163	1.002216395

Acute myocardial infarction || id:ukb-a-533	|| id:eqtl-a-ENSG00000054277	Weighted mode	4	-0.000632278	0.000583554	0.357897163	-0.001776043	0.000511487	0.999367922	0.998225533	1.000511618
Acute myocardial infarction || id:ukb-a-533	|| id:eqtl-a-ENSG00000066230	MR Egger	9	-0.000746762	0.000653271	0.290576233	-0.002027175	0.00053365	0.999253516	0.997974879	1.000533792
Acute myocardial infarction || id:ukb-a-533	|| id:eqtl-a-ENSG00000066230	Weighted median	9	-2.73E-05	0.000401323	0.945674807	-0.000813939	0.000759248	0.999972655	0.999186392	1.000759536
Acute myocardial infarction || id:ukb-a-533	|| id:eqtl-a-ENSG00000066230	Inverse variance weighted	9	-7.71E-05	0.000338376	0.819791677	-0.000740304	0.000586131	0.999922917	0.99925997	1.000586303
Acute myocardial infarction || id:ukb-a-533	|| id:eqtl-a-ENSG00000066230	Simple mode	9	0.000106205	0.000633879	0.87109635	-0.001136196	0.001348607	1.000106211	0.998864449	1.001349517
Acute myocardial infarction || id:ukb-a-533	|| id:eqtl-a-ENSG00000066230	Weighted mode	9	3.08E-05	0.000362071	0.934269538	-0.000678845	0.000740473	1.000030814	0.999321385	1.000740747
Acute myocardial infarction || id:ukb-a-533	|| id:eqtl-a-ENSG00000069667	MR Egger	3	-0.001403814	0.003474908	0.755577514	-0.008214633	0.005407005	0.99859717	0.991819015	1.005421649
Acute myocardial infarction || id:ukb-a-533	|| id:eqtl-a-ENSG00000069667	Weighted median	3	-0.001547173	0.001561998	0.321925415	-0.00460869	0.001514343	0.998454023	0.995401914	1.001515491
Acute myocardial infarction || id:ukb-a-533	|| id:eqtl-a-ENSG00000069667	Inverse variance weighted	3	-0.002104149	0.0014668	0.151424836	-0.004979077	0.000770779	0.997898063	0.995033298	1.000771076
Acute myocardial infarction || id:ukb-a-533	|| id:eqtl-a-ENSG00000069667	Simple mode	3	-0.001634376	0.002323526	0.55466309	-0.006188487	0.002919734	0.998366959	0.993830622	1.002924001
Acute myocardial infarction || id:ukb-a-533	|| id:eqtl-a-ENSG00000069667	Weighted mode	3	-0.001446339	0.001707422	0.48614604	-0.004792885	0.001900207	0.998554706	0.995218582	1.001902014

Acute myocardial infarction || id:ukb-a-533	|| id:eqtl-a-ENSG00000069696	MR Egger	5	-3.49E-05	0.001598931	0.983960613	-0.003168796	0.003099013	0.99996511	0.99683622	1.00310382
Acute myocardial infarction || id:ukb-a-533	|| id:eqtl-a-ENSG00000069696	Weighted median	5	-1.93E-07	0.001140582	0.999864961	-0.002235734	0.002235348	0.999999807	0.997766763	1.002237848
Acute myocardial infarction || id:ukb-a-533	|| id:eqtl-a-ENSG00000069696	Inverse variance weighted	5	0.000190748	0.001055682	0.856612865	-0.001878387	0.002259884	1.000190767	0.998123376	1.00226244
Acute myocardial infarction || id:ukb-a-533	|| id:eqtl-a-ENSG00000069696	Simple mode	5	-0.000217765	0.00192384	0.915331155	-0.003988491	0.003552961	0.999782259	0.996019453	1.00355928
Acute myocardial infarction || id:ukb-a-533	|| id:eqtl-a-ENSG00000069696	Weighted mode	5	-4.54E-05	0.001227793	0.972257626	-0.002451904	0.002361046	0.999954572	0.9975511	1.002363835
Acute myocardial infarction || id:ukb-a-533	|| id:eqtl-a-ENSG00000072310	MR Egger	9	0.001561853	0.000838106	0.104664377	-8.08E-05	0.00320454	1.001563073	0.999919169	1.00320968
Acute myocardial infarction || id:ukb-a-533	|| id:eqtl-a-ENSG00000072310	Weighted median	9	0.0016348	0.000486163	0.000771964	0.00068192	0.002587679	1.001636137	1.000682152	1.00259103
Acute myocardial infarction || id:ukb-a-533	|| id:eqtl-a-ENSG00000072310	Inverse variance weighted	9	0.001459036	0.000399865	0.000263443	0.000675301	0.002242771	1.001460101	1.000675529	1.002245288
Acute myocardial infarction || id:ukb-a-533	|| id:eqtl-a-ENSG00000072310	Simple mode	9	0.001806043	0.000756189	0.043968949	0.000323913	0.003288173	1.001807675	1.000323966	1.003293585
Acute myocardial infarction || id:ukb-a-533	|| id:eqtl-a-ENSG00000072310	Weighted mode	9	0.001683811	0.000475119	0.007575694	0.000752579	0.002615043	1.001685229	1.000752862	1.002618466
Acute myocardial infarction || id:ukb-a-533	|| id:eqtl-a-ENSG00000073614	MR Egger	4	0.001713126	0.002875754	0.611801692	-0.003923352	0.007349603	1.001714594	0.996084335	1.007376678

Acute myocardial infarction || id:ukb-a-533	|| id:eqtl-a-ENSG00000073614	Weighted median	4	-0.000610167	0.000757146	0.420313554	-0.002094172	0.000873839	0.99939002	0.997908019	1.000874221
Acute myocardial infarction || id:ukb-a-533	|| id:eqtl-a-ENSG00000073614	Inverse variance weighted	4	0.000158032	0.000954387	0.868483361	-0.001712566	0.00202863 1	1.000158045	0.9982889	1.00203069
Acute myocardial infarction || id:ukb-a-533	|| id:eqtl-a-ENSG00000073614	Simple mode	4	-0.000942951	0.001561251	0.58847726	-0.004003003	0.00211710 2	0.999057494	0.996004998	1.002119344
Acute myocardial infarction || id:ukb-a-533	|| id:eqtl-a-ENSG00000073614	Weighted mode	4	-0.00046363	0.00110463 8	0.702940954	-0.002628721	0.00170146 1	0.999536478	0.997374731	1.001702909
Acute myocardial infarction || id:ukb-a-533	|| id:eqtl-a-ENSG00000081189	MR Egger	16	-0.003655746	0.00184083 7	0.066984175	-0.007263787	-4.77E-05	0.996350928	0.99276253	0.999952297
Acute myocardial infarction || id:ukb-a-533	|| id:eqtl-a-ENSG00000081189	Weighted median	16	-6.85E-05	0.00067224 1	0.918884728	-0.001386053	0.00124913 2	0.999931542	0.998614907	1.001249913
Acute myocardial infarction || id:ukb-a-533	|| id:eqtl-a-ENSG00000081189	Inverse variance weighted	16	0.001291165	0.00138734 3	0.352022103	-0.001428028	0.00401035 8	1.001291999	0.998572991	1.00401841
Acute myocardial infarction || id:ukb-a-533	|| id:eqtl-a-ENSG00000081189	Simple mode	16	-0.000980167	0.00113434	0.401147923	-0.003203473	0.00124314	0.999020314	0.996801653	1.001243913
Acute myocardial infarction || id:ukb-a-533	|| id:eqtl-a-ENSG00000081189	Weighted mode	16	-0.000189352	0.00066118 2	0.77850133	-0.001485269	0.00110656 4	0.999810666	0.998515834	1.001107177
Acute myocardial infarction || id:ukb-a-533	|| id:eqtl-a-ENSG00000081913	MR Egger	7	0.000488678	0.00119999 9	0.700686034	-0.001863321	0.00284067 7	1.000488798	0.998138414	1.002844715
Acute myocardial infarction || id:ukb-a-533	|| id:eqtl-a-ENSG00000081913	Weighted median	7	0.000443774	0.00060777 2	0.5018385	-0.000851338	0.00173888 6	1.000443873	0.999149024	1.001740399

Acute myocardial infarction || id:ukb-a-533	|| id:eqtl-a-ENSG00000081913	Inverse variance weighted	7	0.000515308	0.000581901	0.375854599	-0.000625218	0.001655834	1.000515441	0.999374978	1.001657206
Acute myocardial infarction || id:ukb-a-533	|| id:eqtl-a-ENSG00000081913	Simple mode	7	0.000772944	0.001257824	0.561442553	-0.00169239	0.00323827 9	1.000773243	0.998309041	1.003243528
Acute myocardial infarction || id:ukb-a-533	|| id:eqtl-a-ENSG00000081913	Weighted mode	7	0.00028866	0.000682192	0.686943008	-0.001048436	0.001625755	1.000288701	0.998952114	1.001627078
Acute myocardial infarction || id:ukb-a-533	|| id:eqtl-a-ENSG00000082014	MR Egger	9	-0.000170658	0.002009209	0.934691775	-0.004108866	0.003767551	0.999829357	0.995899563	1.003774657
Acute myocardial infarction || id:ukb-a-533	|| id:eqtl-a-ENSG00000082014	Weighted median	9	0.000135255	0.001475142	0.926944609	-0.002756023	0.003026534	1.000135264	0.997247771	1.003031118
Acute myocardial infarction || id:ukb-a-533	|| id:eqtl-a-ENSG00000082014	Inverse variance weighted	9	0.000661266	0.001127423	0.557520545	-0.001548484	0.002871016	1.000661484	0.998452714	1.002875141
Acute myocardial infarction || id:ukb-a-533	|| id:eqtl-a-ENSG00000082014	Simple mode	9	0.000829536	0.002344542	0.73262055	-0.003765767	0.00542484	1.000829881	0.996241315	1.005439581
Acute myocardial infarction || id:ukb-a-533	|| id:eqtl-a-ENSG00000082014	Weighted mode	9	0.000351262	0.001939014	0.860751538	-0.003449207	0.00415173	1.000351323	0.996556735	1.0041 6036
Acute myocardial infarction || id:ukb-a-533	|| id:eqtl-a-ENSG00000082701	MR Egger	10	0.001105938	0.001331477	0.430283503	-0.001503757	0.003715633	1.00110655	0.998497373	1.003722544
Acute myocardial infarction || id:ukb-a-533	|| id:eqtl-a-ENSG00000082701	Weighted median	10	0.000607034	0.000546585	0.26674297	-0.000464272	0.00167834	1.000607218	0.999535836	1.001679749
Acute myocardial infarction || id:ukb-a-533	|| id:eqtl-a-ENSG00000082701	Inverse variance weighted	10	0.00056394	0.000447242	0.207334626	-0.000312654	0.001440534	1.000564099	0.999687395	1.001441572

Acute myocardial infarction || id:ukb-a-533	|| id:eqtl-a-ENSG00000082701	Simple mode	10	0.001269187	0.000815575	0.154088126	-0.00032934	0.002867714	1.001269993	0.999670714	1.00287183
Acute myocardial infarction || id:ukb-a-533	|| id:eqtl-a-ENSG00000082701	Weighted mode	10	0.000525954	0.000544375	0.359206637	-0.000541022	0.001592929	1.000526092	0.999459125	1.001594199
Acute myocardial infarction || id:ukb-a-533	|| id:eqtl-a-ENSG00000084676	MR Egger	5	-0.003158381	0.002591875	0.310092244	-0.008238456	0.001921694	0.996846602	0.991795387	1.001923542
Acute myocardial infarction || id:ukb-a-533	|| id:eqtl-a-ENSG00000084676	Weighted median	5	-0.001830858	0.000832417	0.02784599	-0.003462394	-0.000199322	0.998170817	0.996543593	0.999800698
Acute myocardial infarction || id:ukb-a-533	|| id:eqtl-a-ENSG00000084676	Inverse variance weighted	5	-0.00165047	0.000725946	0.02299341	-0.003073325	-0.000227616	0.998350891	0.996931393	0.99977241
Acute myocardial infarction || id:ukb-a-533	|| id:eqtl-a-ENSG00000084676	Simple mode	5	-0.001305066	0.001091156	0.297731404	-0.003443731	0.0008336	0.998695785	0.996562192	1.000833947
Acute myocardial infarction || id:ukb-a-533	|| id:eqtl-a-ENSG00000084676	Weighted mode	5	-0.001856818	0.0009124 7	0.111584305	-0.003645 26	-6.84E-05	0.998144905	0.996361376	0.999931626
Acute myocardial infarction || id:ukb-a-533	|| id:eqtl-a-ENSG00000096717	MR Egger	12	0.001410799	0.000663009	0.059232929	0.000111302	0.00271029 6	1.001411795	1.000111309	1.002713972
Acute myocardial infarction || id:ukb-a-533	|| id:eqtl-a-ENSG00000096717	Weighted median	12	0.000745507	0.000320999	0.020208014	0.00011635	0.001374664	1.000745785	1.000116357	1.00137561
Acute myocardial infarction || id:ukb-a-533	|| id:eqtl-a-ENSG00000096717	Inverse variance weighted	12	0.00073127	0.000310111	0.018369086	0.000123453	0.001339086	1.000731537	1.00012346	1.001339983
Acute myocardial infarction || id:ukb-a-533	|| id:eqtl-a-ENSG00000096717	Simple mode	12	0.001652497	0.000610056	0.020335835	0.000456787	0.002848208	1.001653863	1.000456891	1.002852268

Acute myocardial infarction || id:ukb-a-533	|| id:eqtl-a-ENSG00000096717	Weighted mode	12	0.000780505	0.000286876	0.01990624	0.000218229	0.001342781	1.000780809	1.000218252	1.001343683
Acute myocardial infarction || id:ukb-a-533	|| id:eqtl-a-ENSG00000100104	MR Egger	8	0.001030213	0.001620528	0.548419666	-0.002146022	0.004206447	1.001030744	0.997856279	1.004215307
Acute myocardial infarction || id:ukb-a-533	|| id:eqtl-a-ENSG00000100104	Weighted median	8	0.000242083	0.00092181	0.792845411	-0.001564664	0.002048831	1.000242113	0.998436559	1.002050932
Acute myocardial infarction || id:ukb-a-533	|| id:eqtl-a-ENSG00000100104	Inverse variance weighted	8	0.000242469	0.00083226	0.770792793	-0.001388761	0.001873699	1.000242498	0.998612203	1.001875455
Acute myocardial infarction || id:ukb-a-533	|| id:eqtl-a-ENSG00000100104	Simple mode	8	-0.000285403	0.001614415	0.864684103	-0.003449656	0.00287885	0.999714638	0.996556287	1.002882998
Acute myocardial infarction || id:ukb-a-533	|| id:eqtl-a-ENSG00000100104	Weighted mode	8	0.000289409	0.001023938	0.785625171	-0.001717508	0.002296327	1.000289451	0.998283966	1.002298965
Acute myocardial infarction || id:ukb-a-533	|| id:eqtl-a-ENSG00000100393	MR Egger	6	-0.001348796	0.00176159	0.486555879	-0.004801512	0.002103919	0.998652113	0.995209997	1.002106134
Acute myocardial infarction || id:ukb-a-533	|| id:eqtl-a-ENSG00000100393	Weighted median	6	-0.000561694	0.000602259	0.351003411	-0.001742122	0.000618734	0.999438464	0.998259394	1.000618926
Acute myocardial infarction || id:ukb-a-533	|| id:eqtl-a-ENSG00000100393	Inverse variance weighted	6	-0.000362265	0.000509066	0.476695569	-0.001360035	0.000635504	0.9996378	0.998640889	1.000635706
Acute myocardial infarction || id:ukb-a-533	|| id:eqtl-a-ENSG00000100393	Simple mode	6	-0.000505668	0.000754631	0.532500947	-0.001984746	0.000973409	0.999494446	0.998017223	1.000973883
Acute myocardial infarction || id:ukb-a-533	|| id:eqtl-a-ENSG00000100393	Weighted mode	6	-0.000556513	0.000559479	0.365552986	-0.001653092	0.000540066	0.999443642	0.998348274	1.000540212

Acute myocardial infarction || id:ukb-a-533	|| id:eqtl-a-ENSG00000100462	MR Egger	5	-3.32E-05	0.001445275	0.983101358	-0.002865967	0.002799511	0.999966773	0.997138136	1.002803433
Acute myocardial infarction || id:ukb-a-533	|| id:eqtl-a-ENSG00000100462	Weighted median	5	0.000218058	0.000601043	0.716754658	-0.000959986	0.001396101	1.000218082	0.999040475	1.001397076
Acute myocardial infarction || id:ukb-a-533	|| id:eqtl-a-ENSG00000100462	Inverse variance weighted	5	0.000178415	0.000671972	0.79061738	-0.00113865	0.001495479	1.00017843	0.998861998	1.001496598
Acute myocardial infarction || id:ukb-a-533	|| id:eqtl-a-ENSG00000100462	Simple mode	5	0.001531779	0.00153788	0.37560758	-0.001482466	0.004546025	1.001532953	0.998518632	1.004556374
Acute myocardial infarction || id:ukb-a-533	|| id:eqtl-a-ENSG00000100462	Weighted mode	5	0.000194794	0.000616297	0.767754256	-0.001013149	0.001402737	1.000194813	0.998987364	1.001403721
Acute myocardial infarction || id:ukb-a-533	|| id:eqtl-a-ENSG00000100644	MR Egger	4	0.00055629	0.002669621	0.854228601	-0.004676168	0.005788747	1.000556444	0.995334748	1.005805534
Acute myocardial infarction || id:ukb-a-533	|| id:eqtl-a-ENSG00000100644	Weighted median	4	0.002415595	0.000998748	0.015579365	0.000458049	0.00437314	1.002418514	1.000458154	1.004382716
Acute myocardial infarction || id:ukb-a-533	|| id:eqtl-a-ENSG00000100644	Inverse variance weighted	4	0.002184784	0.000889368	0.014027504	0.000441623	0.003927944	1.002187172	1.00044172	1.003935669
Acute myocardial infarction || id:ukb-a-533	|| id:eqtl-a-ENSG00000100644	Simple mode	4	0.002657927	0.001303936	0.134254811	0.000102213	0.005213642	1.002661463	1.000102218	1.005227257
Acute myocardial infarction || id:ukb-a-533	|| id:eqtl-a-ENSG00000100644	Weighted mode	4	0.002724158	0.001181155	0.104379186	0.000409093	0.005039222	1.002727872	1.000409177	1.00505194
Acute myocardial infarction || id:ukb-a-533	|| id:eqtl-a-ENSG00000101439	MR Egger	21	-0.000188241	0.000579566	0.748884327	-0.00132419	0.000947708	0.999811777	0.998676687	1.000948157

Acute myocardial infarction || id:ukb-a-533	|| id:eqtl-a-ENSG00000101439	Weighted median	21	8.87E-05	0.000380315	0.815653943	-0.000656752	0.000834084	1.00008867	0.999343464	1.000834432
Acute myocardial infarction || id:ukb-a-533	|| id:eqtl-a-ENSG00000101439	Inverse variance weighted	21	0.000229847	0.000410077	0.575140233	-0.000573904	0.001033599	1.000229874	0.99942626	1.001034133
Acute myocardial infarction || id:ukb-a-533	|| id:eqtl-a-ENSG00000101439	Simple mode	21	0.001304902	0.001279874	0.320115299	-0.001203652	0.003813455	1.001305753	0.998797072	1.003820736
Acute myocardial infarction || id:ukb-a-533	|| id:eqtl-a-ENSG00000101439	Weighted mode	21	0.000127852	0.000387014	0.744567472	-0.000630695	0.000886399	1.00012786	0.999369504	1.000886792
Acute myocardial infarction || id:ukb-a-533	|| id:eqtl-a-ENSG00000104856	MR Egger	6	0.000602974	0.003775658	0.880856981	-0.006797317	0.008003264	1.000603155	0.993225733	1.008035376
Acute myocardial infarction || id:ukb-a-533	|| id:eqtl-a-ENSG00000104856	Weighted median	6	0.000779491	0.001382474	0.57286447	-0.001930158	0.00348914	1.000779795	0.998071704	1.003495235
Acute myocardial infarction || id:ukb-a-533	|| id:eqtl-a-ENSG00000104856	Inverse variance weighted	6	0.000455481	0.001204435	0.705304512	-0.001905211	0.002816173	1.000455585	0.998096602	1.002820142
Acute myocardial infarction || id:ukb-a-533	|| id:eqtl-a-ENSG00000104856	Simple mode	6	0.002113731	0.002322576	0.404516285	-0.002438518	0.006665979	1.002115966	0.997564453	1.006688247
Acute myocardial infarction || id:ukb-a-533	|| id:eqtl-a-ENSG00000104856	Weighted mode	6	0.001177849	0.001868427	0.556120429	-0.002484269	0.004839967	1.001178543	0.997518815	1.004851698
Acute myocardial infarction || id:ukb-a-533	|| id:eqtl-a-ENSG00000105835	MR Egger	4	-0.010929194	0.007682407	0.290799402	-0.025986711	0.004128324	0.989130313	0.974348037	1.004136857
Acute myocardial infarction || id:ukb-a-533	|| id:eqtl-a-ENSG00000105835	Weighted median	4	-0.000539841	0.001973729	0.784458764	-0.00440835	0.003328668	0.999460305	0.995601353	1.003334214

Acute myocardial infarction || id:ukb-a-533	|| id:eqtl-a-ENSG00000105835	Inverse variance weighted	4	0.000621646	0.002232544	0.780669086	-0.003754141	0.004997433	1.000621839	0.996252897	1.005009941
Acute myocardial infarction || id:ukb-a-533	|| id:eqtl-a-ENSG00000105835	Simple mode	4	0.004218752	0.003979573	0.366889387	-0.003581211	0.012018716	1.004227664	0.996425194	1.012091231
Acute myocardial infarction || id:ukb-a-533	|| id:eqtl-a-ENSG00000105835	Weighted mode	4	-0.00220868	0.002079779	0.366616015	-0.006285047	0.001867688	0.997793758	0.993734663	1.001869433

Table S2 The statistical results of the hub genes and AMI

gene	P value	fdr_p
NR1H3	<0.0001	0.0005
SREBF1	0.0003	0.00075
NCOA1	0.023	0.023
SIRT1	0.0184	0.023
HIF1A	0.014	0.023

Table S3 BPs in GO items

ONTOLOGY	ID	Description	pvalue	p.adjust	qvalue	geneID	Count
BP	GO:0062013	positive regulation of small molecule metabolic process	2.00E-08	1.82E-05	3.30E-06	NR1H3/SREBF1/SIRT1/HIF1A	4
BP	GO:0030522	intracellular receptor signaling pathway	1.90E-07	3.99E-05	7.25E-06	NR1H3/SREBF1/NCOA1/SIRT1	4

BP	GO:0062012	regulation of small molecule metabolic process	5.01E-07	7.59E-05	1.38E-05	NR1H3/SREBF1/SIRT1/HIF1A	4
BP	GO:0042789	mRNA transcription by RNA polymerase II	1.09E-07	3.99E-05	7.25E-06	NR1H3/SREBF1/NCOA1	3
BP	GO:0009299	mRNA transcription	1.53E-07	3.99E-05	7.25E-06	NR1H3/SREBF1/NCOA1	3
BP	GO:0010883	regulation of lipid storage	2.19E-07	3.99E-05	7.25E-06	NR1H3/SREBF1/SIRT1	3
BP	GO:0019915	lipid storage	9.35E-07	0.000121473	2.21E-05	NR1H3/SREBF1/SIRT1	3
BP	GO:0019217	regulation of fatty acid metabolic process	1.26E-06	0.000143173	2.60E-05	NR1H3/SREBF1/SIRT1	3
BP	GO:0006641	triglyceride metabolic process	1.60E-06	0.000162099	2.95E-05	NR1H3/SREBF1/SIRT1	3
BP	GO:0006639	acylglycerol metabolic process	3.29E-06	0.000258046	4.69E-05	NR1H3/SREBF1/SIRT1	3
BP	GO:0006638	neutral lipid metabolic process	3.37E-06	0.000258046	4.69E-05	NR1H3/SREBF1/SIRT1	3
BP	GO:0010565	regulation of cellular ketone metabolic process	3.60E-06	0.000258046	4.69E-05	NR1H3/SREBF1/SIRT1	3
BP	GO:0046890	regulation of lipid biosynthetic process	8.66E-06	0.000524543	9.54E-05	NR1H3/SREBF1/SIRT1	3
BP	GO:0009755	hormone-mediated signaling pathway	1.02E-05	0.000543429	9.88E-05	NR1H3/NCOA1/SIRT1	3
BP	GO:1901654	response to ketone	1.24E-05	0.000560419	0.000101889	NR1H3/SREBF1/SIRT1	3

BP	GO:0032869	cellular response to insulin stimulus	1.29E-05	0.000560419	0.000101889	SREBF1/NCOA1/SIRT1	3
BP	GO:0007623	circadian rhythm	1.33E-05	0.000560419	0.000101889	NR1H3/SREBF1/SIRT1	3
BP	GO:0042180	cellular ketone metabolic process	1.49E-05	0.000560419	0.000101889	NR1H3/SREBF1/SIRT1	3
BP	GO:0031669	cellular response to nutrient levels	1.77E-05	0.000618515	0.000112451	SREBF1/NCOA1/SIRT1	3
BP	GO:0050708	regulation of protein secretion	2.43E-05	0.000737626	0.000134106	NR1H3/SREBF1/HIF1A	3
BP	GO:0031668	cellular response to extracellular stimulus	2.58E-05	0.000756177	0.000137479	SREBF1/NCOA1/SIRT1	3
BP	GO:0032868	response to insulin	2.79E-05	0.000792638	0.000144108	SREBF1/NCOA1/SIRT1	3
BP	GO:0061448	connective tissue development	2.95E-05	0.000812121	0.00014765	NCOA1/SIRT1/HIF1A	3
BP	GO:0048511	rhythmic process	3.94E-05	0.000962959	0.000175073	NR1H3/SREBF1/SIRT1	3
BP	GO:0071375	cellular response to peptide hormone stimulus	4.10E-05	0.000962959	0.000175073	SREBF1/NCOA1/SIRT1	3
BP	GO:0071496	cellular response to external stimulus	5.04E-05	0.001042214	0.000189482	SREBF1/NCOA1/SIRT1	3
BP	GO:0048545	response to steroid hormone	5.57E-05	0.001073351	0.000195143	NR1H3/SREBF1/SIRT1	3
BP	GO:0051235	maintenance of location	5.71E-05	0.001073351	0.000195143	NR1H3/SREBF1/SIRT1	3

BP	GO:0010506	regulation of autophagy	5.81E-05	0.001073351	0.000195143	SREBF1/SIRT1/HIF1A	3
BP	GO:0019216	regulation of lipid metabolic process	5.97E-05	0.001073351	0.000195143	NR1H3/SREBF1/SIRT1	3
BP	GO:0009306	protein secretion	6.60E-05	0.001100451	0.00020007	NR1H3/SREBF1/HIF1A	3
BP	GO:0035592	establishment of protein localization to extracellular region	6.66E-05	0.001100451	0.00020007	NR1H3/SREBF1/HIF1A	3
BP	GO:0071692	protein localization to extracellular region	7.11E-05	0.001121175	0.0002038 38	NR1H3/SREBF1/HIF1A	3
BP	GO:1901653	cellular response to peptide	7.40E-05	0.001121175	0.000203838	SREBF1/NCOA1/SIRT1	3
BP	GO:0046486	glycerolipid metabolic process	9.04E-05	0.001274259	0.00023167	NR1H3/SREBF1/SIRT1	3
BP	GO:0006631	fatty acid metabolic process	9.11E-05	0.001274259	0.00023167	NR1H3/SREBF1/SIRT1	3
BP	GO:0043434	response to peptide hormone	0.000106097	0.001397708	0.000254114	SREBF1/NCOA1/SIRT1	3
BP	GO:0016570	histone modification	0.000150897	0.001773508	0.000322437	SREBF1/NCOA1/SIRT1	3
BP	GO:0031667	response to nutrient levels	0.000150897	0.001773508	0.000322437	SREBF1/NCOA1/SIRT1	3
BP	GO:0010876	lipid localization	0.000159559	0.001812994	0.000329616	NR1H3/SREBF1/SIRT1	3
BP	GO:0019221	cytokine-mediated signaling pathway	0.00017264	0.001913774	0.000347939	NR1H3/SIRT1/HIF1A	3

BP	GO:1904177	regulation of adipose tissue development	3.69E-06	0.000258046	4.69E-05	NCOA1/SIRT1	2
BP	GO:0010867	positive regulation of triglyceride biosynthetic process	5.87E-06	0.000381083	6.93E-05	NR1H3/SREBF1	2
BP	GO:1902176	negative regulation of oxidative stress-induced intrinsic apoptotic signaling pathway	9.55E-06	0.000542813	9.87E-05	SIRT1/HIF1A	2
BP	GO:0010866	regulation of triglyceride biosynthetic process	1.41E-05	0.000560419	0.000101889	NR1H3/SREBF1	2
BP	GO:0031065	positive regulation of histone deacetylation	1.41E-05	0.000560419	0.000101889	SREBF1/SIRT1	2
BP	GO:0035357	peroxisome proliferator activated receptor signaling pathway	1.54E-05	0.000560419	0.000101889	NCOA1/SIRT1	2
BP	GO:0090208	positive regulation of triglyceride metabolic process	1.54E-05	0.000560419	0.000101889	NR1H3/SREBF1	2
BP	GO:0010875	positive regulation of cholesterol efflux	1.96E-05	0.000659704	0.000119939	NR1H3/SIRT1	2
BP	GO:0090312	positive regulation of protein deacetylation	2.27E-05	0.000710301	0.000129138	SREBF1/SIRT1	2

BP	GO:1902175	regulation of oxidative stress-induced intrinsic apoptotic signaling pathway	2.27E-05	0.000710301	0.000129138	SIRT1/HIF1A	2
BP	GO:1903146	regulation of autophagy of mitochondrion	3.51E-05	0.000939407	0.000170791	SREBF1/HIF1A	2
BP	GO:0032373	positive regulation of sterol transport	4.13E-05	0.000962959	0.000175073	NR1H3/SIRT1	2
BP	GO:0032376	positive regulation of cholesterol transport	4.13E-05	0.000962959	0.000175073	NR1H3/SIRT1	2
BP	GO:0032570	response to progesterone	4.13E-05	0.000962959	0.000175073	NR1H3/SREBF1	2
BP	GO:0019432	triglyceride biosynthetic process	4.57E-05	0.001038652	0.000188835	NR1H3/SREBF1	2
BP	GO:0055090	acylglycerol homeostasis	4.80E-05	0.001038652	0.000188835	NR1H3/SIRT1	2
BP	GO:0070328	triglyceride homeostasis	4.80E-05	0.001038652	0.000188835	NR1H3/SIRT1	2
BP	GO:0090207	regulation of triglyceride metabolic process	5.03E-05	0.001042214	0.000189482	NR1H3/SREBF1	2
BP	GO:0031063	regulation of histone deacetylation	5.27E-05	0.001064885	0.000193604	SREBF1/SIRT1	2
BP	GO:0008631	intrinsic apoptotic signaling pathway in response to oxidative stress	5.52E-05	0.001073351	0.000195143	SIRT1/HIF1A	2

BP	GO:1902895	positive regulation of miRNA transcription	6.02E-05	0.001073351	0.000195143	SREBF1/HIF1A	2
BP	GO:0046460	neutral lipid biosynthetic process	6.55E-05	0.001100451	0.00020007	NR1H3/SREBF1	2
BP	GO:0046463	acylglycerol biosynthetic process	6.55E-05	0.001100451	0.00020007	NR1H3/SREBF1	2
BP	GO:0042304	regulation of fatty acid biosynthetic process	6.82E-05	0.001107379	0.00020133	NR1H3/SIRT1	2
BP	GO:0032007	negative regulation of TOR signaling	7.38E-05	0.001121175	0.000203838	SIRT1/HIF1A	2
BP	GO:0060612	adipose tissue development	7.38E-05	0.001121175	0.000203838	NCOA1/SIRT1	2
BP	GO:0010874	regulation of cholesterol efflux	7.67E-05	0.001143219	0.000207846	NR1H3/SIRT1	2
BP	GO:1903202	negative regulation of oxidative stress-induced cell death	8.27E-05	0.001211861	0.000220326	SIRT1/HIF1A	2
BP	GO:2000630	positive regulation of miRNA metabolic process	8.57E-05	0.001236665	0.000224835	SREBF1/HIF1A	2
BP	GO:0090311	regulation of protein deacetylation	0.000101796	0.001381078	0.000251091	SREBF1/SIRT1	2
BP	GO:1902893	regulation of miRNA transcription	0.000101796	0.001381078	0.000251091	SREBF1/HIF1A	2
BP	GO:0061614	miRNA transcription	0.000105178	0.001397708	0.000254114	SREBF1/HIF1A	2

BP	GO:0033344	cholesterol efflux	0.000138084	0.001773508	0.000322437	NR1H3/SIRT1	2
BP	GO:0050709	negative regulation of protein secretion	0.000142014	0.001773508	0.000322437	NR1H3/SREBF1	2
BP	GO:1900076	regulation of cellular response to insulin stimulus	0.000142014	0.001773508	0.000322437	NCOA1/SIRT1	2
BP	GO:0043967	histone H4 acetylation	0.000150039	0.001773508	0.000322437	NCOA1/SIRT1	2
BP	GO:2000628	regulation of miRNA metabolic process	0.000150039	0.001773508	0.000322437	SREBF1/HIF1A	2
BP	GO:0016239	positive regulation of macroautophagy	0.000154133	0.001773508	0.000322437	SIRT1/HIF1A	2
BP	GO:0043536	positive regulation of blood vessel endothelial cell migration	0.000154133	0.001773508	0.000322437	SIRT1/HIF1A	2
BP	GO:0050810	regulation of steroid biosynthetic process	0.000154133	0.001773508	0.000322437	SREBF1/SIRT1	2
BP	GO:0045913	positive regulation of carbohydrate metabolic process	0.000166744	0.00187124	0.000340206	SIRT1/HIF1A	2
BP	GO:1903201	regulation of oxidative stress-induced cell death	0.000175425	0.001921215	0.000349292	SIRT1/HIF1A	2
BP	GO:0032371	regulation of sterol transport	0.000179847	0.00192233	0.000349671	NR1H3/SIRT1	2

BP	GO:0032374	regulation of cholesterol transport	0.000179847	0.0019233	0.000349671	NR1H3/SIRT1	2
BP	GO:1905897	regulation of response to endoplasmic reticulum stress	0.000184323	0.001948253	0.000354207	NR1H3/SIRT1	2
BP	GO:0001892	embryonic placenta development	0.000207524	0.002168269	0.000394208	NCOA1/HIF1A	2
BP	GO:0032370	positive regulation of lipid transport	0.000212328	0.002168605	0.000394269	NR1H3/SIRT1	2
BP	GO:0046889	positive regulation of lipid biosynthetic process	0.000212328	0.002168605	0.000394269	NR1H3/SREBF1	2
BP	GO:0007589	body fluid secretion	0.000217186	0.002193576	0.000398809	NR1H3/HIF1A	2
BP	GO:0010586	miRNA metabolic process	0.000222098	0.002218542	0.000403348	SREBF1/HIF1A	2
BP	GO:0000422	autophagy of mitochondrion	0.000237162	0.002293408	0.000416959	SREBF1/HIF1A	2
BP	GO:0061726	mitochondrion disassembly	0.000237162	0.002293408	0.000416959	SREBF1/HIF1A	2
BP	GO:2000106	regulation of leukocyte apoptotic process	0.000237162	0.002293408	0.000416959	SIRT1/HIF1A	2
BP	GO:1900407	regulation of cellular response to oxidative stress	0.000247476	0.002367958	0.000430513	SIRT1/HIF1A	2
BP	GO:0036473	cell death in response to oxidative stress	0.000274212	0.002542407	0.000462229	SIRT1/HIF1A	2

BP	GO:0042632	cholesterol homeostasis	0.000274212	0.002542407	0.000462229	NR1H3/SIRT1	2
BP	GO:0055092	sterol homeostasis	0.000279722	0.002542407	0.000462229	NR1H3/SIRT1	2
BP	GO:2001243	negative regulation of intrinsic apoptotic signaling pathway	0.000279722	0.002542407	0.000462229	SIRT1/HIF1A	2
BP	GO:0016575	histone deacetylation	0.000285287	0.002542407	0.000462229	SREBF1/SIRT1	2
BP	GO:0019218	regulation of steroid metabolic process	0.000285287	0.002542407	0.000462229	SREBF1/SIRT1	2
BP	GO:0031058	positive regulation of histone modification	0.000285287	0.002542407	0.000462229	SREBF1/SIRT1	2
BP	GO:1902882	regulation of response to oxidative stress	0.000296577	0.002617368	0.000475858	SIRT1/HIF1A	2
BP	GO:0032006	regulation of TOR signaling	0.000319809	0.002795255	0.000508199	SIRT1/HIF1A	2
BP	GO:1905954	positive regulation of lipid localization	0.000350065	0.003030567	0.00055098	NR1H3/SIRT1	2
BP	GO:0001938	positive regulation of endothelial cell proliferation	0.000362546	0.003109005	0.000565241	SIRT1/HIF1A	2
BP	GO:0071887	leukocyte apoptotic process	0.000375243	0.00318781	0.000579568	SIRT1/HIF1A	2
BP	GO:0030301	cholesterol transport	0.000401283	0.003370966	0.000612867	NR1H3/SIRT1	2
BP	GO:0006476	protein deacetylation	0.000407928	0.003370966	0.000612867	SREBF1/SIRT1	2

BP	GO:0008286	insulin receptor signaling pathway	0.000407928	0.003370966	0.000612867	SREBF1/SIRT1	2
BP	GO:0051224	negative regulation of protein transport	0.000455948	0.003733841	0.000678841	NR1H3/SREBF1	2
BP	GO:0031929	TOR signaling	0.000463023	0.003757924	0.000683219	SIRT1/HIF1A	2
BP	GO:0010595	positive regulation of endothelial cell migration	0.000470151	0.003782014	0.000687599	SIRT1/HIF1A	2
BP	GO:0035601	protein deacylation	0.00048457	0.003830208	0.000696361	SREBF1/SIRT1	2
BP	GO:1904950	negative regulation of establishment of protein localization	0.00048457	0.003830208	0.000696361	NR1H3/SREBF1	2
BP	GO:0015918	sterol transport	0.000499203	0.003911857	0.000711206	NR1H3/SIRT1	2
BP	GO:0098732	macromolecule deacylation	0.00051405	0.003993777	0.000726099	SREBF1/SIRT1	2
BP	GO:1903008	organelle disassembly	0.000529112	0.004075959	0.000741041	SREBF1/HIF1A	2
BP	GO:0010508	positive regulation of autophagy	0.000567704	0.004336495	0.000788408	SIRT1/HIF1A	2
BP	GO:0050796	regulation of insulin secretion	0.000607632	0.004537562	0.000824963	SREBF1/HIF1A	2
BP	GO:0001890	placenta development	0.000615778	0.004537562	0.000824963	NCOA1/HIF1A	2
BP	GO:0010821	regulation of mitochondrion organization	0.000615778	0.004537562	0.000824963	SREBF1/HIF1A	2

BP	GO:0043535	regulation of blood vessel endothelial cell migration	0.000615778	0.004537562	0.000824963	SIRT1/HIF1A	2
BP	GO:0071456	cellular response to hypoxia	0.000623977	0.004537562	0.000824963	SIRT1/HIF1A	2
BP	GO:1903531	negative regulation of secretion by cell	0.000623977	0.004537562	0.000824963	NR1H3/SREBF1	2
BP	GO:0045834	positive regulation of lipid metabolic process	0.000648895	0.004681312	0.000851098	NR1H3/SREBF1	2
BP	GO:0032368	regulation of lipid transport	0.000657307	0.004704663	0.000855344	NR1H3/SIRT1	2
BP	GO:0001678	cellular glucose homeostasis	0.000682864	0.004849401	0.000881658	SIRT1/HIF1A	2
BP	GO:0036294	cellular response to decreased oxygen levels	0.000691489	0.004872588	0.000885874	SIRT1/HIF1A	2
BP	GO:0016241	regulation of macroautophagy	0.000700168	0.004895791	0.000890092	SIRT1/HIF1A	2
BP	GO:0006633	fatty acid biosynthetic process	0.000735414	0.005102989	0.000927762	NR1H3/SIRT1	2
BP	GO:0001959	regulation of cytokine-mediated signaling pathway	0.000762407	0.005250209	0.000954528	NR1H3/HIF1A	2
BP	GO:2001242	regulation of intrinsic apoptotic signaling pathway	0.000808455	0.005511438	0.001002022	SIRT1/HIF1A	2

BP	GO:0055088	lipid homeostasis	0.000827245	0.005511438	0.001002022	NR1H3/SIRT1	2
BP	GO:0009267	cellular response to starvation	0.00083672	0.005511438	0.001002022	SREBF1/SIRT1	2
BP	GO:0031056	regulation of histone modification	0.00083672	0.005511438	0.001002022	SREBF1/SIRT1	2
BP	GO:0051048	negative regulation of secretion	0.00083672	0.005511438	0.001002022	NR1H3/SREBF1	2
BP	GO:0071453	cellular response to oxygen levels	0.00083672	0.005511438	0.001002022	SIRT1/HIF1A	2
BP	GO:0010634	positive regulation of epithelial cell migration	0.000846247	0.005534093	0.00100614	SIRT1/HIF1A	2
BP	GO:0043534	blood vessel endothelial cell migration	0.000855828	0.005556768	0.001010263	SIRT1/HIF1A	2
BP	GO:0006694	steroid biosynthetic process	0.000865461	0.005563	0.001011396	SREBF1/SIRT1	2
BP	GO:0060759	regulation of response to cytokine stimulus	0.000875147	0.005563	0.001011396	NR1H3/HIF1A	2
BP	GO:0090276	regulation of peptide hormone secretion	0.000875147	0.005563	0.001011396	SREBF1/HIF1A	2
BP	GO:0016573	histone acetylation	0.000884886	0.005585845	0.001015549	NCOA1/SIRT1	2
BP	GO:0002791	regulation of peptide secretion	0.000904523	0.005654477	0.001028027	SREBF1/HIF1A	2
BP	GO:0001936	regulation of endothelial cell proliferation	0.00091442	0.005654477	0.001028027	SIRT1/HIF1A	2

BP	GO:0030073	insulin secretion	0.00091442	0.005654477	0.001028027	SREBF1/HIF1A	2
BP	GO:0090087	regulation of peptide transport	0.000924371	0.005671079	0.001031046	SREBF1/HIF1A	2
BP	GO:1905952	regulation of lipid localization	0.000934374	0.005671079	0.001031046	NR1H3/SIRT1	2
BP	GO:0006109	regulation of carbohydrate metabolic process	0.00094443	0.005671079	0.001031046	SIRT1/HIF1A	2
BP	GO:0018393	internal peptidyl-lysine acetylation	0.00094443	0.005671079	0.001031046	NCOA1/SIRT1	2
BP	GO:0045766	positive regulation of angiogenesis	0.000954538	0.005671079	0.001031046	SIRT1/HIF1A	2
BP	GO:1904018	positive regulation of vasculature development	0.000954538	0.005671079	0.001031046	SIRT1/HIF1A	2
BP	GO:0006475	internal protein amino acid acetylation	0.000964699	0.005694232	0.001035255	NCOA1/SIRT1	2
BP	GO:0009749	response to glucose	0.001026773	0.006021527	0.00109476	SREBF1/HIF1A	2
BP	GO:0018394	peptidyl-lysine acetylation	0.00105852	0.006167916	0.001121374	NCOA1/SIRT1	2
BP	GO:0001935	endothelial cell proliferation	0.001079948	0.006213117	0.001129592	SIRT1/HIF1A	2
BP	GO:0009746	response to hexose	0.001079948	0.006213117	0.001129592	SREBF1/HIF1A	2
BP	GO:1903828	negative regulation of protein localization	0.001123433	0.006422644	0.001167686	NR1H3/SREBF1	2

BP	GO:0000302	response to reactive oxygen species	0.001134435	0.00644501	0.001171752	SIRT1/HIF1A	2
BP	GO:0034284	response to monosaccharide	0.001167757	0.006593111	0.001198678	SREBF1/HIF1A	2
BP	GO:0042594	response to starvation	0.001212919	0.006805823	0.001237351	SREBF1/SIRT1	2
BP	GO:0072330	monocarboxylic acid biosynthetic process	0.001293966	0.007172041	0.001303932	NR1H3/SIRT1	2
BP	GO:1901215	negative regulation of neuron death	0.001293966	0.007172041	0.001303932	SIRT1/HIF1A	2
BP	GO:0050679	positive regulation of epithelial cell proliferation	0.00136547	0.007522496	0.001367647	SIRT1/HIF1A	2
BP	GO:0030072	peptide hormone secretion	0.001377569	0.007543437	0.001371455	SREBF1/HIF1A	2
BP	GO:0002790	peptide secretion	0.001438849	0.007739132	0.001407034	SREBF1/HIF1A	2
BP	GO:0006473	protein acetylation	0.001438849	0.007739132	0.001407034	NCOA1/SIRT1	2
BP	GO:0010594	regulation of endothelial cell migration	0.001438849	0.007739132	0.001407034	SIRT1/HIF1A	2
BP	GO:2001234	negative regulation of apoptotic signaling pathway	0.00147624	0.007868176	0.001430495	SIRT1/HIF1A	2
BP	GO:0009743	response to carbohydrate	0.001488808	0.007868176	0.001430495	SREBF1/HIF1A	2
BP	GO:0046883	regulation of hormone secretion	0.001488808	0.007868176	0.001430495	SREBF1/HIF1A	2

BP	GO:0045444	fat cell differentiation	0.001617337	0.00849803	0.001545007	SREBF1/SIRT1	2
BP	GO:1901617	organic hydroxy compound biosynthetic process	0.001630475	0.008517824	0.001548606	SREBF1/SIRT1	2
BP	GO:0042593	glucose homeostasis	0.001670198	0.00864599	0.001571907	SIRT1/HIF1A	2
BP	GO:0033500	carbohydrate homeostasis	0.001683543	0.00864599	0.001571907	SIRT1/HIF1A	2
BP	GO:0045926	negative regulation of growth	0.001683543	0.00864599	0.001571907	SIRT1/HIF1A	2
BP	GO:0015833	peptide transport	0.00175104	0.008892152	0.001616661	SREBF1/HIF1A	2
BP	GO:0045017	glycerolipid biosynthetic process	0.00175104	0.008892152	0.001616661	NR1H3/SREBF1	2
BP	GO:0034976	response to endoplasmic reticulum stress	0.001847701	0.009330892	0.001696428	NR1H3/SIRT1	2
BP	GO:0043543	protein acylation	0.002004689	0.010067749	0.001830394	NCOA1/SIRT1	2
BP	GO:0043542	endothelial cell migration	0.002137748	0.010676996	0.00194116	SIRT1/HIF1A	2
BP	GO:0034599	cellular response to oxidative stress	0.002244102	0.011146931	0.002026597	SIRT1/HIF1A	2
BP	GO:0046879	hormone secretion	0.002290447	0.011269631	0.002048905	SREBF1/HIF1A	2
BP	GO:0010632	regulation of epithelial cell migration	0.002305997	0.011269631	0.002048905	SIRT1/HIF1A	2
BP	GO:0015850	organic hydroxy compound transport	0.002305997	0.011269631	0.002048905	NR1H3/SIRT1	2

BP	GO:0001666	response to hypoxia	0.002368707	0.011452951	0.002082234	SIRT1/HIF1A	2
BP	GO:0042886	amide transport	0.002368707	0.011452951	0.002082234	SREBF1/HIF1A	2
BP	GO:0097193	intrinsic apoptotic signaling pathway	0.002400367	0.011544625	0.002098901	SIRT1/HIF1A	2
BP	GO:0009914	hormone transport	0.002432231	0.011636305	0.002115569	SREBF1/HIF1A	2
BP	GO:0036293	response to decreased oxygen levels	0.002578129	0.011832963	0.002151323	SIRT1/HIF1A	2
BP	GO:0016236	macroautophagy	0.00271126	0.012064024	0.002193332	SIRT1/HIF1A	2
BP	GO:0009416	response to light stimulus	0.002779038	0.012064024	0.002193332	SIRT1/HIF1A	2
BP	GO:0008202	steroid metabolic process	0.002813229	0.012064024	0.002193332	SREBF1/SIRT1	2
BP	GO:0031647	regulation of protein stability	0.002847622	0.012064024	0.002193332	SREBF1/SIRT1	2
BP	GO:1901214	regulation of neuron death	0.002847622	0.012064024	0.002193332	SIRT1/HIF1A	2
BP	GO:0046394	carboxylic acid biosynthetic process	0.002864894	0.012064024	0.002193332	NR1H3/SIRT1	2
BP	GO:0016053	organic acid biosynthetic process	0.00289959	0.012064024	0.002193332	NR1H3/SIRT1	2
BP	GO:0070482	response to oxygen levels	0.003058207	0.012635956	0.002297313	SIRT1/HIF1A	2
BP	GO:0090287	regulation of cellular response to growth factor stimulus	0.003076082	0.012639912	0.002298033	SIRT1/HIF1A	2
BP	GO:0062197	cellular response to	0.003202612	0.01271255	0.002311239	SIRT1/HIF1A	2

		chemical stress					
BP	GO:0045765	regulation of angiogenesis	0.003276015	0.012947382	0.002353933	SIRT1/HIF1A	2
BP	GO:1901342	regulation of vasculature development	0.003387619	0.0130072	0.002364808	SIRT1/HIF1A	2
BP	GO:0010631	epithelial cell migration	0.003596885	0.013566674	0.002466525	SIRT1/HIF1A	2
BP	GO:0070997	neuron death	0.00363558	0.013609333	0.002474281	SIRT1/HIF1A	2
BP	GO:0090132	epithelium migration	0.003655002	0.013609333	0.002474281	SIRT1/HIF1A	2
BP	GO:0090130	tissue migration	0.003752855	0.013700182	0.002490798	SIRT1/HIF1A	2
BP	GO:2001233	regulation of apoptotic signaling pathway	0.003752855	0.013700182	0.002490798	SIRT1/HIF1A	2
BP	GO:0050878	regulation of body fluid levels	0.004074299	0.014081891	0.002560196	NR1H3/HIF1A	2
BP	GO:0001701	in utero embryonic development	0.004135976	0.014240916	0.002589107	NCOA1/HIF1A	2
BP	GO:0030099	myeloid cell differentiation	0.004429652	0.014731163	0.002678238	SIRT1/HIF1A	2
BP	GO:0018205	peptidyl-lysine modification	0.004580094	0.014922241	0.002712978	NCOA1/SIRT1	2
BP	GO:0050678	regulation of epithelial cell proliferation	0.004580094	0.014922241	0.002712978	SIRT1/HIF1A	2
BP	GO:0031331	positive regulation of cellular catabolic process	0.004910526	0.015373742	0.002795064	SIRT1/HIF1A	2
BP	GO:0006869	lipid transport	0.005000491	0.015373742	0.002795064	NR1H3/SIRT1	2

BP	GO:0006979	response to oxidative stress	0.005023104	0.015373742	0.002795064	SIRT1/HIF1A	2
BP	GO:0048568	embryonic organ development	0.005368111	0.015998731	0.002908692	NCOA1/HIF1A	2
BP	GO:0009314	response to radiation	0.005438418	0.016097522	0.002926653	SIRT1/HIF1A	2
BP	GO:0023061	signal release	0.005869364	0.016365802	0.002975428	SREBF1/HIF1A	2
BP	GO:0051051	negative regulation of transport	0.006090668	0.016576099	0.003013662	NR1H3/SREBF1	2
BP	GO:0050673	epithelial cell proliferation	0.006140373	0.016661489	0.003029186	SIRT1/HIF1A	2
BP	GO:0022411	cellular component disassembly	0.006190269	0.01673397	0.003042364	SREBF1/HIF1A	2
BP	GO:0001819	positive regulation of cytokine production	0.006265471	0.01673397	0.003042364	SIRT1/HIF1A	2
BP	GO:0001667	ameboidal-type cell migration	0.006417162	0.016907826	0.003073972	SIRT1/HIF1A	2
BP	GO:0001542	ovulation from ovarian follicle	0.002642565	0.011832963	0.002151323	SIRT1	1
BP	GO:0032070	regulation of deoxyribonuclease activity	0.002642565	0.011832963	0.002151323	SIRT1	1
BP	GO:0033034	positive regulation of myeloid cell apoptotic process	0.002642565	0.011832963	0.002151323	SIRT1	1
BP	GO:0033483	gas homeostasis	0.002642565	0.011832963	0.002151323	HIF1A	1
BP	GO:0045542	positive regulation of cholesterol	0.002642565	0.011832963	0.002151323	SREBF1	1

		biosynthetic process					
BP	GO:0046886	positive regulation of hormone biosynthetic process	0.002642565	0.011832963	0.002151323	HIF1A	1
BP	GO:0051006	positive regulation of lipoprotein lipase activity	0.002642565	0.011832963	0.002151323	NR1H3	1
BP	GO:0070099	regulation of chemokine-mediated signaling pathway	0.002642565	0.011832963	0.002151323	HIF1A	1
BP	GO:0106120	positive regulation of sterol biosynthetic process	0.002642565	0.011832963	0.002151323	SREBF1	1
BP	GO:1902237	positive regulation of endoplasmic reticulum stress-induced intrinsic apoptotic signaling pathway	0.002642565	0.011832963	0.002151323	SIRT1	1
BP	GO:1990700	nucleolar chromatin organization	0.002642565	0.011832963	0.002151323	SIRT1	1
BP	GO:2001054	negative regulation of mesenchymal cell apoptotic process	0.002642565	0.011832963	0.002151323	HIF1A	1
BP	GO:0002069	columnar/cuboidal epithelial cell maturation	0.002906514	0.012064024	0.002193332	HIF1A	1

BP	GO:0010887	negative regulation of cholesterol storage	0.002906514	0.012064024	0.002193332	NR1H3	1
BP	GO:0033210	leptin-mediated signaling pathway	0.002906514	0.012064024	0.002193332	SIRT1	1
BP	GO:0051574	positive regulation of histone H3-K9 methylation	0.002906514	0.012064024	0.002193332	SIRT1	1
BP	GO:0061365	positive regulation of triglyceride lipase activity	0.002906514	0.012064024	0.002193332	NR1H3	1
BP	GO:0061418	regulation of transcription from RNA polymerase II promoter in response to hypoxia	0.002906514	0.012064024	0.002193332	HIF1A	1
BP	GO:0071394	cellular response to testosterone stimulus	0.002906514	0.012064024	0.002193332	SIRT1	1
BP	GO:2000109	regulation of macrophage apoptotic process	0.002906514	0.012064024	0.002193332	SIRT1	1
BP	GO:2000480	negative regulation of cAMP-dependent protein kinase activity	0.002906514	0.012064024	0.002193332	SIRT1	1
BP	GO:0000012	single strand break repair	0.003170407	0.012639912	0.002298033	SIRT1	1
BP	GO:0034145	positive regulation of toll-like	0.003170407	0.012639912	0.002298033	NR1H3	1

		receptor 4 signaling pathway					
BP	GO:0048548	regulation of pinocytosis	0.003170407	0.012639912	0.002298033	NR1H3	1
BP	GO:0051095	regulation of helicase activity	0.003170407	0.012639912	0.002298033	SIRT1	1
BP	GO:0051573	negative regulation of histone H3-K9 methylation	0.003170407	0.012639912	0.002298033	SIRT1	1
BP	GO:0070857	regulation of bile acid biosynthetic process	0.003170407	0.012639912	0.002298033	SIRT1	1
BP	GO:1900112	regulation of histone H3-K9 trimethylation	0.003170407	0.012639912	0.002298033	SIRT1	1
BP	GO:0010745	negative regulation of macrophage derived foam cell differentiation	0.003434244	0.0130072	0.002364808	NR1H3	1
BP	GO:0030157	pancreatic juice secretion	0.003434244	0.0130072	0.002364808	NR1H3	1
BP	GO:0031392	regulation of prostaglandin biosynthetic process	0.003434244	0.0130072	0.002364808	SIRT1	1
BP	GO:0042541	hemoglobin biosynthetic process	0.003434244	0.0130072	0.002364808	HIF1A	1
BP	GO:0070243	regulation of thymocyte apoptotic process	0.003434244	0.0130072	0.002364808	HIF1A	1

BP	GO:0070914	UV-damage excision repair	0.003434244	0.0130072	0.002364808	SIRT1	1
BP	GO:0070932	histone H3 deacetylation	0.003434244	0.0130072	0.002364808	SIRT1	1
BP	GO:1903599	positive regulation of autophagy of mitochondrion	0.003434244	0.0130072	0.002364808	HIF1A	1
BP	GO:2001053	regulation of mesenchymal cell apoptotic process	0.003434244	0.0130072	0.002364808	HIF1A	1
BP	GO:0036151	phosphatidylcholine acyl-chain remodeling	0.003698025	0.013609333	0.002474281	NR1H3	1
BP	GO:0045348	positive regulation of MHC class II biosynthetic process	0.003698025	0.013609333	0.002474281	SIRT1	1
BP	GO:0071888	macrophage apoptotic process	0.003698025	0.013609333	0.002474281	SIRT1	1
BP	GO:0090239	regulation of histone H4 acetylation	0.003698025	0.013609333	0.002474281	SIRT1	1
BP	GO:0032352	positive regulation of hormone metabolic process	0.003961751	0.013745159	0.002498975	HIF1A	1
BP	GO:0032933	SREBP signaling pathway	0.003961751	0.013745159	0.002498975	SREBF1	1
BP	GO:0035358	regulation of peroxisome proliferator activated receptor signaling pathway	0.003961751	0.013745159	0.002498975	SIRT1	1

BP	GO:0043518	negative regulation of DNA damage response, signal transduction by p53 class mediator	0.003961751	0.013745159	0.002498975	SIRT1	1
BP	GO:0060330	regulation of response to interferon-gamma	0.003961751	0.013745159	0.002498975	NR1H3	1
BP	GO:0060334	regulation of interferon-gamma-mediated signaling pathway	0.003961751	0.013745159	0.002498975	NR1H3	1
BP	GO:0060576	intestinal epithelial cell development	0.003961751	0.013745159	0.002498975	HIF1A	1
BP	GO:0060766	negative regulation of androgen receptor signaling pathway	0.003961751	0.013745159	0.002498975	SIRT1	1
BP	GO:0090205	positive regulation of cholesterol metabolic process	0.003961751	0.013745159	0.002498975	SREBF1	1
BP	GO:0097152	mesenchymal cell apoptotic process	0.003961751	0.013745159	0.002498975	HIF1A	1
BP	GO:1902166	negative regulation of intrinsic apoptotic signaling pathway in response to DNA damage by p53 class mediator	0.003961751	0.013745159	0.002498975	SIRT1	1

BP	GO:1904251	regulation of bile acid metabolic process	0.003961751	0.013745159	0.002498975	SIRT1	1
BP	GO:2001279	regulation of unsaturated fatty acid biosynthetic process	0.003961751	0.013745159	0.002498975	SIRT1	1
BP	GO:0050872	white fat cell differentiation	0.00422542	0.014439501	0.002625212	SIRT1	1
BP	GO:0071501	cellular response to sterol depletion	0.00422542	0.014439501	0.002625212	SREBF1	1
BP	GO:0006089	lactate metabolic process	0.004489034	0.014731163	0.002678238	HIF1A	1
BP	GO:0007000	nucleolus organization	0.004489034	0.014731163	0.002678238	SIRT1	1
BP	GO:0020027	hemoglobin metabolic process	0.004489034	0.014731163	0.002678238	HIF1A	1
BP	GO:0030949	positive regulation of vascular endothelial growth factor receptor signaling pathway	0.004489034	0.014731163	0.002678238	HIF1A	1
BP	GO:0036124	histone H3-K9 trimethylation	0.004489034	0.014731163	0.002678238	SIRT1	1
BP	GO:0055089	fatty acid homeostasis	0.004489034	0.014731163	0.002678238	SIRT1	1
BP	GO:0060457	negative regulation of digestive system process	0.004489034	0.014731163	0.002678238	NR1H3	1

BP	GO:0090594	inflammatory response to wounding	0.004489034	0.014731163	0.002678238	HIF1A	1
BP	GO:1900034	regulation of cellular response to heat	0.004489034	0.014731163	0.002678238	SIRT1	1
BP	GO:1902165	regulation of intrinsic apoptotic signaling pathway in response to DNA damage by p53 class mediator	0.004489034	0.014731163	0.002678238	SIRT1	1
BP	GO:0006991	response to sterol depletion	0.004752592	0.015158268	0.002755889	SREBF1	1
BP	GO:0019896	axonal transport of mitochondrion	0.004752592	0.015158268	0.002755889	HIF1A	1
BP	GO:0035067	negative regulation of histone acetylation	0.004752592	0.015158268	0.002755889	SIRT1	1
BP	GO:0044320	cellular response to leptin stimulus	0.004752592	0.015158268	0.002755889	SIRT1	1
BP	GO:0045722	positive regulation of gluconeogenesis	0.004752592	0.015158268	0.002755889	SIRT1	1
BP	GO:0060644	mammary gland epithelial cell differentiation	0.004752592	0.015158268	0.002755889	HIF1A	1
BP	GO:0010885	regulation of cholesterol storage	0.005016094	0.015373742	0.002795064	NR1H3	1

BP	GO:0036003	positive regulation of transcription from RNA polymerase II promoter in response to stress	0.005016094	0.015373742	0.002795064	HIF1A	1
BP	GO:0043031	negative regulation of macrophage activation	0.005016094	0.015373742	0.002795064	NR1H3	1
BP	GO:0045346	regulation of MHC class II biosynthetic process	0.005016094	0.015373742	0.002795064	SIRT1	1
BP	GO:0051152	positive regulation of smooth muscle cell differentiation	0.005016094	0.015373742	0.002795064	SIRT1	1
BP	GO:0061298	retina vasculature development in camera-type eye	0.005016094	0.015373742	0.002795064	HIF1A	1
BP	GO:0070242	thymocyte apoptotic process	0.005016094	0.015373742	0.002795064	HIF1A	1
BP	GO:2000434	regulation of protein neddylation	0.005016094	0.015373742	0.002795064	HIF1A	1
BP	GO:2000774	positive regulation of cellular senescence	0.005016094	0.015373742	0.002795064	SIRT1	1
BP	GO:0033762	response to glucagon	0.005279541	0.015786521	0.00287011	SREBF1	1
BP	GO:0045342	MHC class II biosynthetic process	0.005279541	0.015786521	0.00287011	SIRT1	1
BP	GO:0051004	regulation of lipoprotein	0.005279541	0.015786521	0.00287011	NR1H3	1

		lipase activity					
BP	GO:0060749	mammary gland alveolus development	0.005279541	0.015786521	0.00287011	HIF1A	1
BP	GO:0061377	mammary gland lobule development	0.005279541	0.015786521	0.00287011	HIF1A	1
BP	GO:0097709	connective tissue replacement	0.005279541	0.015786521	0.00287011	HIF1A	1
BP	GO:2000479	regulation of cAMP-dependent protein kinase activity	0.005279541	0.015786521	0.00287011	SIRT1	1
BP	GO:0010878	cholesterol storage	0.0055429 31	0.016097522	0.002926653	NR1H3	1
BP	GO:0030502	negative regulation of bone mineralization	0.005542931	0.016097522	0.002926653	HIF1A	1
BP	GO:0043984	histone H4-K16 acetylation	0.005542931	0.016097522	0.002926653	SIRT1	1
BP	GO:0045821	positive regulation of glycolytic process	0.005542931	0.016097522	0.002926653	HIF1A	1
BP	GO:1901522	positive regulation of transcription from RNA polymerase II promoter involved in cellular response to chemical stimulus	0.005542931	0.016097522	0.002926653	HIF1A	1

BP	GO:1903204	negative regulation of oxidative stress-induced neuron death	0.005542931	0.016097522	0.002926653	HIF1A	1
BP	GO:2000757	negative regulation of peptidyl-lysine acetylation	0.005542931	0.016097522	0.002926653	SIRT1	1
BP	GO:0002070	epithelial cell maturation	0.005806266	0.016239678	0.002952498	HIF1A	1
BP	GO:0006907	pinocytosis	0.005806266	0.016239678	0.002952498	NR1H3	1
BP	GO:0010888	negative regulation of lipid storage	0.005806266	0.016239678	0.002952498	NR1H3	1
BP	GO:0030728	ovulation	0.005806266	0.016239678	0.002952498	SIRT1	1
BP	GO:0032069	regulation of nuclease activity	0.005806266	0.016239678	0.002952498	SIRT1	1
BP	GO:0032780	negative regulation of ATP-dependent activity	0.005806266	0.016239678	0.002952498	SIRT1	1
BP	GO:0044154	histone H3-K14 acetylation	0.005806266	0.016239678	0.002952498	SIRT1	1
BP	GO:0044321	response to leptin	0.005806266	0.016239678	0.002952498	SIRT1	1
BP	GO:0045717	negative regulation of fatty acid biosynthetic process	0.005806266	0.016239678	0.002952498	SIRT1	1
BP	GO:0046628	positive regulation of insulin receptor signaling pathway	0.005806266	0.016239678	0.002952498	SIRT1	1

BP	GO:0051000	positive regulation of nitric-oxide synthase activity	0.005806266	0.016239678	0.002952498	HIF1A	1
BP	GO:0060713	labyrinthine layer morphogenesis	0.005806266	0.016239678	0.002952498	NCOA1	1
BP	GO:0010893	positive regulation of steroid biosynthetic process	0.006069545	0.016568216	0.003012229	SREBF1	1
BP	GO:0031061	negative regulation of histone methylation	0.006069545	0.016568216	0.003012229	SIRT1	1
BP	GO:0045540	regulation of cholesterol biosynthetic process	0.006069545	0.016568216	0.003012229	SREBF1	1
BP	GO:0045723	positive regulation of fatty acid biosynthetic process	0.006069545	0.016568216	0.003012229	NR1H3	1
BP	GO:0090335	regulation of brown fat cell differentiation	0.006069545	0.016568216	0.003012229	SIRT1	1
BP	GO:0106118	regulation of sterol biosynthetic process	0.006069545	0.016568216	0.003012229	SREBF1	1
BP	GO:1902254	negative regulation of intrinsic apoptotic signaling pathway by p53 class mediator	0.006069545	0.016568216	0.003012229	SIRT1	1

BP	GO:0006346	DNA methylation-dependent heterochromatin formation	0.006332768	0.01673397	0.003042364	SIRT1	1
BP	GO:0006925	inflammatory cell apoptotic process	0.006332768	0.01673397	0.003042364	SIRT1	1
BP	GO:0014850	response to muscle activity	0.006332768	0.01673397	0.003042364	HIF1A	1
BP	GO:0035162	embryonic hemopoiesis	0.006332768	0.01673397	0.003042364	HIF1A	1
BP	GO:0060571	morphogenesis of an epithelial fold	0.006332768	0.01673397	0.003042364	HIF1A	1
BP	GO:0060907	positive regulation of macrophage cytokine production	0.006332768	0.01673397	0.003042364	SIRT1	1
BP	GO:0070233	negative regulation of T cell apoptotic process	0.006332768	0.01673397	0.003042364	HIF1A	1
BP	GO:0046716	muscle cell cellular homeostasis	0.006595935	0.017130585	0.003114472	HIF1A	1
BP	GO:0046885	regulation of hormone biosynthetic process	0.006595935	0.017130585	0.003114472	HIF1A	1
BP	GO:0060575	intestinal epithelial cell differentiation	0.006595935	0.017130585	0.003114472	HIF1A	1
BP	GO:1900078	positive regulation of cellular response to insulin stimulus	0.006595935	0.017130585	0.003114472	SIRT1	1

BP	GO:1901984	negative regulation of protein acetylation	0.006595935	0.017130585	0.003114472	SIRT1	1
BP	GO:0034643	establishment of mitochondrion localization, microtubule-mediated	0.006859047	0.017513689	0.003184123	HIF1A	1
BP	GO:0047497	mitochondrion transport along microtubule	0.006859047	0.017513689	0.003184123	HIF1A	1
BP	GO:0051570	regulation of histone H3-K9 methylation	0.006859047	0.017513689	0.003184123	SIRT1	1
BP	GO:0060333	interferon-gamma-mediated signaling pathway	0.006859047	0.017513689	0.003184123	NR1H3	1
BP	GO:0060669	embryonic placenta morphogenesis	0.006859047	0.017513689	0.003184123	NCOA1	1
BP	GO:2000773	negative regulation of cellular senescence	0.006859047	0.017513689	0.003184123	SIRT1	1
BP	GO:0034143	regulation of toll-like receptor 4 signaling pathway	0.007122102	0.017983309	0.003269503	NR1H3	1
BP	GO:0035774	positive regulation of insulin secretion involved in cellular response to	0.007122102	0.017983309	0.003269503	HIF1A	1

		glucose stimulus					
BP	GO:0045116	protein neddylation	0.007122102	0.017983309	0.003269503	HIF1A	1
BP	GO:1902932	positive regulation of alcohol biosynthetic process	0.007122102	0.017983309	0.003269503	SREBF1	1
BP	GO:0002052	positive regulation of neuroblast proliferation	0.007385102	0.018493273	0.003362219	HIF1A	1
BP	GO:0060765	regulation of androgen receptor signaling pathway	0.007385102	0.018493273	0.003362219	SIRT1	1
BP	GO:1900017	positive regulation of cytokine production involved in inflammatory response	0.007385102	0.018493273	0.003362219	HIF1A	1
BP	GO:0010575	positive regulation of vascular endothelial growth factor production	0.007648047	0.018994739	0.003453389	HIF1A	1
BP	GO:0034123	positive regulation of toll-like receptor signaling pathway	0.007648047	0.018994739	0.003453389	NR1H3	1
BP	GO:0051654	establishment of mitochondrion localization	0.007648047	0.018994739	0.003453389	HIF1A	1
BP	GO:0045940	positive regulation of steroid	0.007910935	0.019382858	0.003523952	SREBF1	1

		metabolic process					
BP	GO:0070168	negative regulation of biomineral tissue development	0.007910935	0.019382858	0.003523952	HIF1A	1
BP	GO:1902230	negative regulation of intrinsic apoptotic signaling pathway in response to DNA damage	0.007910935	0.019382858	0.003523952	SIRT1	1
BP	GO:1903203	regulation of oxidative stress-induced neuron death	0.007910935	0.019382858	0.003523952	HIF1A	1
BP	GO:2000108	positive regulation of leukocyte apoptotic process	0.007910935	0.019382858	0.003523952	SIRT1	1
BP	GO:0010039	response to iron ion	0.008173768	0.019919452	0.003621509	HIF1A	1
BP	GO:0110150	negative regulation of biomineralization	0.008173768	0.019919452	0.003621509	HIF1A	1
BP	GO:0001516	prostaglandin biosynthetic process	0.008436546	0.020075445	0.00364987	SIRT1	1
BP	GO:0010743	regulation of macrophage derived foam cell differentiation	0.008436546	0.020075445	0.00364987	NR1H3	1
BP	GO:0032770	positive regulation of monooxygenase activity	0.008436546	0.020075445	0.00364987	HIF1A	1

BP	GO:0034390	smooth muscle cell apoptotic process	0.008436546	0.020075445	0.00364987	SIRT1	1
BP	GO:0034391	regulation of smooth muscle cell apoptotic process	0.008436546	0.020075445	0.00364987	SIRT1	1
BP	GO:0046457	prostanoid biosynthetic process	0.008436546	0.020075445	0.00364987	SIRT1	1
BP	GO:0140718	facultative heterochromatin formation	0.008436546	0.020075445	0.00364987	SIRT1	1
BP	GO:1902235	regulation of endoplasmic reticulum stress-induced intrinsic apoptotic signaling pathway	0.008436546	0.020075445	0.00364987	SIRT1	1
BP	GO:1903715	regulation of aerobic respiration	0.008436546	0.020075445	0.00364987	HIF1A	1
BP	GO:0001782	B cell homeostasis	0.008699267	0.020433162	0.003714905	HIF1A	1
BP	GO:0030947	regulation of vascular endothelial growth factor receptor signaling pathway	0.008699267	0.020433162	0.003714905	HIF1A	1
BP	GO:0033032	regulation of myeloid cell apoptotic process	0.008699267	0.020433162	0.003714905	SIRT1	1
BP	GO:0036475	neuron death in response to oxidative stress	0.008699267	0.020433162	0.003714905	HIF1A	1

BP	GO:1902253	regulation of intrinsic apoptotic signaling pathway by p53 class mediator	0.008699267	0.020433162	0.003714905	SIRT1	1
BP	GO:0045648	positive regulation of erythrocyte differentiation	0.008961933	0.020888197	0.003797634	HIF1A	1
BP	GO:0061081	positive regulation of myeloid leukocyte cytokine production involved in immune response	0.008961933	0.020888197	0.003797634	SIRT1	1
BP	GO:1901797	negative regulation of signal transduction by p53 class mediator	0.008961933	0.020888197	0.003797634	SIRT1	1
BP	GO:0046676	negative regulation of insulin secretion	0.009224543	0.021282004	0.003869231	SREBF1	1
BP	GO:0051567	histone H3-K9 methylation	0.009224543	0.021282004	0.003869231	SIRT1	1
BP	GO:0071398	cellular response to fatty acid	0.009224543	0.021282004	0.003869231	SREBF1	1
BP	GO:1905898	positive regulation of response to endoplasmic reticulum stress	0.009224543	0.021282004	0.003869231	SIRT1	1

BP	GO:0006699	bile acid biosynthetic process	0.009487097	0.021777201	0.003959262	SIRT1	1
BP	GO:1903580	positive regulation of ATP metabolic process	0.009487097	0.021777201	0.003959262	HIF1A	1
BP	GO:0002068	glandular epithelial cell development	0.009749596	0.022100706	0.004018077	HIF1A	1
BP	GO:0010934	macrophage cytokine production	0.009749596	0.022100706	0.004018077	SIRT1	1
BP	GO:0010935	regulation of macrophage cytokine production	0.009749596	0.022100706	0.004018077	SIRT1	1
BP	GO:0033144	negative regulation of intracellular steroid hormone receptor signaling pathway	0.009749596	0.022100706	0.004018077	SIRT1	1
BP	GO:0070229	negative regulation of lymphocyte apoptotic process	0.009749596	0.022100706	0.004018077	HIF1A	1
BP	GO:0010742	macrophage derived foam cell differentiation	0.010012039	0.022471466	0.004085485	NR1H3	1
BP	GO:0032094	response to food	0.010012039	0.022471466	0.004085485	SREBF1	1
BP	GO:0043516	regulation of DNA damage response, signal transduction by p53 class mediator	0.010012039	0.022471466	0.004085485	SIRT1	1

BP	GO:1902229	regulation of intrinsic apoptotic signaling pathway in response to DNA damage	0.010012039	0.022471466	0.004085485	SIRT1	1
BP	GO:0030279	negative regulation of ossification	0.010274427	0.02261369	0.004111342	HIF1A	1
BP	GO:0032350	regulation of hormone metabolic process	0.010274427	0.02261369	0.004111342	HIF1A	1
BP	GO:0033028	myeloid cell apoptotic process	0.010274427	0.02261369	0.004111342	SIRT1	1
BP	GO:0043618	regulation of transcription from RNA polymerase II promoter in response to stress	0.010274427	0.02261369	0.004111342	HIF1A	1
BP	GO:0045922	negative regulation of fatty acid metabolic process	0.010274427	0.02261369	0.004111342	SIRT1	1
BP	GO:0071634	regulation of transforming growth factor beta production	0.010274427	0.02261369	0.004111342	HIF1A	1
BP	GO:0090077	foam cell differentiation	0.010274427	0.02261369	0.004111342	NR1H3	1
BP	GO:0090181	regulation of cholesterol metabolic process	0.010274427	0.02261369	0.004111342	SREBF1	1
BP	GO:0000731	DNA synthesis involved in DNA repair	0.010536759	0.022913669	0.00416588	SIRT1	1

BP	GO:0032941	secretion by tissue	0.010536759	0.022913669	0.00416588	NR1H3	1
BP	GO:0045923	positive regulation of fatty acid metabolic process	0.010536759	0.022913669	0.00416588	NR1H3	1
BP	GO:0090278	negative regulation of peptide hormone secretion	0.010536759	0.022913669	0.00416588	SREBF1	1
BP	GO:1902692	regulation of neuroblast proliferation	0.010536759	0.022913669	0.00416588	HIF1A	1
BP	GO:0002792	negative regulation of peptide secretion	0.010799035	0.023372197	0.004249244	SREBF1	1
BP	GO:0070232	regulation of T cell apoptotic process	0.010799035	0.023372197	0.004249244	HIF1A	1
BP	GO:0010907	positive regulation of glucose metabolic process	0.011061255	0.023602538	0.004291122	SIRT1	1
BP	GO:0031670	cellular response to nutrient	0.011061255	0.023602538	0.004291122	NCOA1	1
BP	GO:0034142	toll-like receptor 4 signaling pathway	0.011061255	0.023602538	0.004291122	NR1H3	1
BP	GO:0050999	regulation of nitric-oxide synthase activity	0.011061255	0.023602538	0.004291122	HIF1A	1
BP	GO:0071542	dopaminergic neuron differentiation	0.011061255	0.023602538	0.004291122	HIF1A	1

BP	GO:0071604	transforming growth factor beta production	0.011061255	0.023602538	0.004291122	HIF1A	1
BP	GO:0007595	lactation	0.01132342	0.023937184	0.004351963	HIF1A	1
BP	GO:0033574	response to testosterone	0.01132342	0.023937184	0.004351963	SIRT1	1
BP	GO:0051150	regulation of smooth muscle cell differentiation	0.01132342	0.023937184	0.004351963	SIRT1	1
BP	GO:0097009	energy homeostasis	0.01132342	0.023937184	0.004351963	SIRT1	1
BP	GO:0042771	intrinsic apoptotic signaling pathway in response to DNA damage by p53 class mediator	0.01158553	0.024265545	0.004411662	SIRT1	1
BP	GO:0044058	regulation of digestive system process	0.01158553	0.024265545	0.004411662	NR1H3	1
BP	GO:1903214	regulation of protein targeting to mitochondrion	0.01158553	0.024265545	0.004411662	SREBF1	1
BP	GO:1903573	negative regulation of response to endoplasmic reticulum stress	0.01158553	0.024265545	0.004411662	NR1H3	1
BP	GO:0043620	regulation of DNA-templated transcription in response to stress	0.011847584	0.024700581	0.004490755	HIF1A	1

BP	GO:0062208	positive regulation of pattern recognition receptor signaling pathway	0.011847584	0.024700581	0.004490755	NR1H3	1
BP	GO:2000273	positive regulation of signaling receptor activity	0.012109582	0.025158201	0.004573953	HIF1A	1
BP	GO:0006110	regulation of glycolytic process	0.012371524	0.025158201	0.004573953	HIF1A	1
BP	GO:0006984	ER-nucleus signaling pathway	0.012371524	0.025158201	0.004573953	SREBF1	1
BP	GO:0008542	visual learning	0.012371524	0.025158201	0.004573953	HIF1A	1
BP	GO:0010824	regulation of centrosome duplication	0.012371524	0.025158201	0.004573953	SIRT1	1
BP	GO:0030521	androgen receptor signaling pathway	0.012371524	0.025158201	0.004573953	SIRT1	1
BP	GO:0032369	negative regulation of lipid transport	0.012371524	0.025158201	0.004573953	NR1H3	1
BP	GO:0045646	regulation of erythrocyte differentiation	0.012371524	0.025158201	0.004573953	HIF1A	1
BP	GO:0060711	labyrinthine layer development	0.012371524	0.025158201	0.004573953	NCOA1	1
BP	GO:0061647	histone H3-K9 modification	0.012371524	0.025158201	0.004573953	SIRT1	1
BP	GO:0120163	negative regulation of cold-induced	0.012371524	0.025158201	0.004573953	NR1H3	1

		thermogenesis					
BP	GO:0031057	negative regulation of histone modification	0.012633411	0.025462907	0.004629351	SIRT1	1
BP	GO:0031648	protein destabilization	0.012633411	0.025462907	0.004629351	SIRT1	1
BP	GO:0045981	positive regulation of nucleotide metabolic process	0.012633411	0.025462907	0.004629351	HIF1A	1
BP	GO:1900544	positive regulation of purine nucleotide metabolic process	0.012633411	0.025462907	0.004629351	HIF1A	1
BP	GO:0008206	bile acid metabolic process	0.012895243	0.025705649	0.004673484	SIRT1	1
BP	GO:0043457	regulation of cellular respiration	0.012895243	0.025705649	0.004673484	HIF1A	1
BP	GO:0048546	digestive tract morphogenesis	0.012895243	0.025705649	0.004673484	HIF1A	1
BP	GO:0061178	regulation of insulin secretion involved in cellular response to glucose stimulus	0.012895243	0.025705649	0.004673484	HIF1A	1
BP	GO:2000378	negative regulation of reactive oxygen species metabolic process	0.012895243	0.025705649	0.004673484	HIF1A	1

BP	GO:0002066	columnar/cuboidal epithelial cell development	0.013157019	0.025886862	0.00470643	HIF1A	1
BP	GO:0006692	prostanoid metabolic process	0.013157019	0.025886862	0.00470643	SIRT1	1
BP	GO:0006693	prostaglandin metabolic process	0.013157019	0.025886862	0.00470643	SIRT1	1
BP	GO:0051646	mitochondrion localization	0.013157019	0.025886862	0.00470643	HIF1A	1
BP	GO:0061082	myeloid leukocyte cytokine production	0.013157019	0.025886862	0.00470643	SIRT1	1
BP	GO:1903747	regulation of establishment of protein localization to mitochondrion	0.013157019	0.025886862	0.00470643	SREBF1	1
BP	GO:0022602	ovulation cycle process	0.013418739	0.026063321	0.004738511	SIRT1	1
BP	GO:0030225	macrophage differentiation	0.013418739	0.026063321	0.004738511	SIRT1	1
BP	GO:0031062	positive regulation of histone methylation	0.013418739	0.026063321	0.004738511	SIRT1	1
BP	GO:0043124	negative regulation of I-kappaB kinase/NF-kappaB signaling	0.013418739	0.026063321	0.004738511	SIRT1	1
BP	GO:0043277	apoptotic cell clearance	0.013418739	0.026063321	0.004738511	NR1H3	1
BP	GO:0050873	brown fat cell differentiation	0.013418739	0.026063321	0.004738511	SIRT1	1

BP	GO:0006636	unsaturated fatty acid biosynthetic process	0.013680404	0.026458484	0.004810355	SIRT1	1
BP	GO:1902930	regulation of alcohol biosynthetic process	0.013680404	0.026458484	0.004810355	SREBF1	1
BP	GO:0006111	regulation of gluconeogenesis	0.013942014	0.026850191	0.00488157	SIRT1	1
BP	GO:0007632	visual behavior	0.013942014	0.026850191	0.00488157	HIF1A	1
BP	GO:0042149	cellular response to glucose starvation	0.014203567	0.027238487	0.004952165	SIRT1	1
BP	GO:0046605	regulation of centrosome cycle	0.014203567	0.027238487	0.004952165	SIRT1	1
BP	GO:2000107	negative regulation of leukocyte apoptotic process	0.014465066	0.027565503	0.005011619	HIF1A	1
BP	GO:2000179	positive regulation of neural precursor cell proliferation	0.014465066	0.027565503	0.005011619	HIF1A	1
BP	GO:2000772	regulation of cellular senescence	0.014465066	0.027565503	0.005011619	SIRT1	1
BP	GO:0010676	positive regulation of cellular carbohydrate metabolic process	0.014726509	0.027888326	0.005070311	SIRT1	1
BP	GO:0043470	regulation of carbohydrate catabolic process	0.014726509	0.027888326	0.005070311	HIF1A	1

BP	GO:0045806	negative regulation of endocytosis	0.014726509	0.027888326	0.005070311	NR1H3	1
BP	GO:0018023	peptidyl-lysine trimethylation	0.014987896	0.028207034	0.005128255	SIRT1	1
BP	GO:0032768	regulation of monooxygenase activity	0.014987896	0.028207034	0.005128255	HIF1A	1
BP	GO:0070231	T cell apoptotic process	0.014987896	0.028207034	0.005128255	HIF1A	1
BP	GO:0001961	positive regulation of cytokine-mediated signaling pathway	0.015249228	0.028404812	0.005164212	HIF1A	1
BP	GO:0006695	cholesterol biosynthetic process	0.015249228	0.028404812	0.005164212	SREBF1	1
BP	GO:0048010	vascular endothelial growth factor receptor signaling pathway	0.015249228	0.028404812	0.005164212	HIF1A	1
BP	GO:0051898	negative regulation of protein kinase B signaling	0.015249228	0.028404812	0.005164212	SIRT1	1
BP	GO:1902653	secondary alcohol biosynthetic process	0.015249228	0.028404812	0.005164212	SREBF1	1
BP	GO:0001755	neural crest cell migration	0.015510504	0.028714966	0.005220601	HIF1A	1
BP	GO:0010574	regulation of vascular endothelial	0.015510504	0.028714966	0.005220601	HIF1A	1

		growth factor production					
BP	GO:0035773	insulin secretion involved in cellular response to glucose stimulus	0.015510504	0.028714966	0.005220601	HIF1A	1
BP	GO:0045599	negative regulation of fat cell differentiation	0.015771725	0.029080118	0.005286988	SIRT1	1
BP	GO:0051353	positive regulation of oxidoreductase activity	0.015771725	0.029080118	0.005286988	HIF1A	1
BP	GO:0046456	icosanoid biosynthetic process	0.016032891	0.029442217	0.00535282	SIRT1	1
BP	GO:0090497	mesenchymal cell migration	0.016032891	0.029442217	0.00535282	HIF1A	1
BP	GO:0043030	regulation of macrophage activation	0.016294001	0.029741459	0.005407225	NR1H3	1
BP	GO:0046888	negative regulation of hormone secretion	0.016294001	0.029741459	0.005407225	SREBF1	1
BP	GO:0070228	regulation of lymphocyte apoptotic process	0.016294001	0.029741459	0.005407225	HIF1A	1
BP	GO:0010573	vascular endothelial growth factor production	0.016555055	0.029917585	0.005439246	HIF1A	1
BP	GO:0051055	negative regulation of lipid biosynthetic process	0.016555055	0.029917585	0.005439246	SIRT1	1

BP	GO:0070059	intrinsic apoptotic signaling pathway in response to endoplasmic reticulum stress	0.016555055	0.029917585	0.005439246	SIRT1	1
BP	GO:0070542	response to fatty acid	0.016555055	0.029917585	0.005439246	SREBF1	1
BP	GO:2001244	positive regulation of intrinsic apoptotic signaling pathway	0.016555055	0.029917585	0.005439246	SIRT1	1
BP	GO:0001947	heart looping	0.016816055	0.030268898	0.005503117	HIF1A	1
BP	GO:0098930	axonal transport	0.016816055	0.030268898	0.005503117	HIF1A	1
BP	GO:0016126	sterol biosynthetic process	0.017076998	0.030497036	0.005544595	SREBF1	1
BP	GO:0035065	regulation of histone acetylation	0.017076998	0.030497036	0.005544595	SIRT1	1
BP	GO:0060760	positive regulation of response to cytokine stimulus	0.017076998	0.030497036	0.005544595	HIF1A	1
BP	GO:1905953	negative regulation of lipid localization	0.017076998	0.030497036	0.005544595	NR1H3	1
BP	GO:0006879	cellular iron ion homeostasis	0.017337887	0.030841759	0.005607268	HIF1A	1
BP	GO:0034605	cellular response to heat	0.017337887	0.030841759	0.005607268	SIRT1	1
BP	GO:0007405	neuroblast proliferation	0.01759872	0.031244602	0.005680508	HIF1A	1

BP	GO:0032024	positive regulation of insulin secretion	0.017859497	0.031522879	0.005731101	HIF1A	1
BP	GO:0042446	hormone biosynthetic process	0.017859497	0.031522879	0.005731101	HIF1A	1
BP	GO:0060193	positive regulation of lipase activity	0.017859497	0.031522879	0.005731101	NR1H3	1
BP	GO:0002260	lymphocyte homeostasis	0.018120219	0.031797837	0.00578109	HIF1A	1
BP	GO:0061180	mammary gland epithelium development	0.018120219	0.031797837	0.00578109	HIF1A	1
BP	GO:0061371	determination of heart left/right asymmetry	0.018120219	0.031797837	0.00578109	HIF1A	1
BP	GO:0003143	embryonic heart tube morphogenesis	0.018380886	0.032069531	0.005830486	HIF1A	1
BP	GO:0014823	response to activity	0.018380886	0.032069531	0.005830486	HIF1A	1
BP	GO:0032722	positive regulation of chemokine production	0.018380886	0.032069531	0.005830486	HIF1A	1
BP	GO:0003208	cardiac ventricle morphogenesis	0.018641498	0.03239985	0.005890541	HIF1A	1
BP	GO:0032922	circadian regulation of gene expression	0.018641498	0.03239985	0.005890541	SIRT1	1
BP	GO:0033143	regulation of intracellular steroid hormone receptor	0.018902054	0.032665336	0.005938808	SIRT1	1

		signaling pathway					
BP	GO:0042698	ovulation cycle	0.018902054	0.032665336	0.005938808	SIRT1	1
BP	GO:0046626	regulation of insulin receptor signaling pathway	0.018902054	0.032665336	0.005938808	SIRT1	1
BP	GO:0002067	glandular epithelial cell differentiation	0.019162555	0.032927717	0.005986511	HIF1A	1
BP	GO:0043462	regulation of ATP-dependent activity	0.019162555	0.032927717	0.005986511	SIRT1	1
BP	GO:1903578	regulation of ATP metabolic process	0.019162555	0.032927717	0.005986511	HIF1A	1
BP	GO:0030330	DNA damage response, signal transduction by p53 class mediator	0.019423	0.033124778	0.006022338	SIRT1	1
BP	GO:0051145	smooth muscle cell differentiation	0.019423	0.033124778	0.006022338	SIRT1	1
BP	GO:0051298	centrosome duplication	0.019423	0.033124778	0.006022338	SIRT1	1
BP	GO:2000756	regulation of peptidyl-lysine acetylation	0.019423	0.033124778	0.006022338	SIRT1	1
BP	GO:0071479	cellular response to ionizing radiation	0.01968339	0.033505995	0.006091646	SIRT1	1
BP	GO:0010822	positive regulation of mitochondrio	0.019943725	0.033885693	0.006160678	HIF1A	1

		n organization					
BP	GO:0002534	cytokine production involved in inflammatory response	0.020204004	0.033947209	0.006171862	HIF1A	1
BP	GO:0008088	axo-dendritic transport	0.020204004	0.033947209	0.006171862	HIF1A	1
BP	GO:0034121	regulation of toll-like receptor signaling pathway	0.020204004	0.033947209	0.006171862	NR1H3	1
BP	GO:0045824	negative regulation of innate immune response	0.020204004	0.033947209	0.006171862	NR1H3	1
BP	GO:0046470	phosphatidylcholine metabolic process	0.020204004	0.033947209	0.006171862	NR1H3	1
BP	GO:1900015	regulation of cytokine production involved in inflammatory response	0.020204004	0.033947209	0.006171862	HIF1A	1
BP	GO:0043966	histone H3 acetylation	0.020464228	0.034257797	0.00622833	SIRT1	1
BP	GO:1903533	regulation of protein targeting	0.020464228	0.034257797	0.00622833	SREBF1	1
BP	GO:0002720	positive regulation of cytokine production involved in immune response	0.020724397	0.034629554	0.006295918	SIRT1	1
BP	GO:0003151	outflow tract morphogenesis	0.020984511	0.034871884	0.006339975	HIF1A	1

BP	GO:0014068	positive regulation of phosphatidylinositol 3-kinase signaling	0.020984511	0.034871884	0.006339975	SIRT1	1
BP	GO:0070227	lymphocyte apoptotic process	0.020984511	0.034871884	0.006339975	HIF1A	1
BP	GO:0031060	regulation of histone methylation	0.021244569	0.035175434	0.006395163	SIRT1	1
BP	GO:0072332	intrinsic apoptotic signaling pathway by p53 class mediator	0.021244569	0.035175434	0.006395163	SIRT1	1
BP	GO:0051149	positive regulation of muscle cell differentiation	0.021504572	0.03547669	0.006449934	SIRT1	1
BP	GO:2001021	negative regulation of response to DNA damage stimulus	0.021504572	0.03547669	0.006449934	SIRT1	1
BP	GO:0006094	gluconeogenesis	0.02176452	0.035582641	0.006469197	SIRT1	1
BP	GO:0006096	glycolytic process	0.02176452	0.035582641	0.006469197	HIF1A	1
BP	GO:0014032	neural crest cell development	0.02176452	0.035582641	0.006469197	HIF1A	1
BP	GO:0030500	regulation of bone mineralization	0.02176452	0.035582641	0.006469197	HIF1A	1
BP	GO:0031507	heterochromatin formation	0.02176452	0.035582641	0.006469197	SIRT1	1
BP	GO:0006289	nucleotide-excision repair	0.022024412	0.035814295	0.006511313	SIRT1	1

BP	GO:0006757	ATP generation from ADP	0.022024412	0.035814295	0.006511313	HIF1A	1
BP	GO:0035050	embryonic heart tube development	0.022024412	0.035814295	0.006511313	HIF1A	1
BP	GO:0008306	associative learning	0.02228425	0.036172113	0.006576367	HIF1A	1
BP	GO:0019319	hexose biosynthetic process	0.022544032	0.036528565	0.006641173	SIRT1	1
BP	GO:0010660	regulation of muscle cell apoptotic process	0.022803759	0.036883659	0.006705732	SIRT1	1
BP	GO:0055072	iron ion homeostasis	0.02306343	0.03710559	0.00674608	HIF1A	1
BP	GO:0072384	organelle transport along microtubule	0.02306343	0.03710559	0.00674608	HIF1A	1
BP	GO:1901983	regulation of protein acetylation	0.02306343	0.03710559	0.00674608	SIRT1	1
BP	GO:0001960	negative regulation of cytokine-mediated signaling pathway	0.023323047	0.037325087	0.006785987	NR1H3	1
BP	GO:0034644	cellular response to UV	0.023323047	0.037325087	0.006785987	SIRT1	1
BP	GO:0048864	stem cell development	0.023323047	0.037325087	0.006785987	HIF1A	1
BP	GO:0046364	monosaccharide biosynthetic process	0.023582608	0.037411153	0.006801634	SIRT1	1
BP	GO:0051591	response to cAMP	0.023582608	0.037411153	0.006801634	SREBF1	1

BP	GO:0060191	regulation of lipase activity	0.023582608	0.037411153	0.006801634	NR1H3	1
BP	GO:0070098	chemokine-mediated signaling pathway	0.023582608	0.037411153	0.006801634	HIF1A	1
BP	GO:2000177	regulation of neural precursor cell proliferation	0.023582608	0.037411153	0.006801634	HIF1A	1
BP	GO:0070828	heterochromatin organization	0.023842114	0.037756937	0.00686645	SIRT1	1
BP	GO:0010657	muscle cell apoptotic process	0.024101565	0.037969363	0.006903121	SIRT1	1
BP	GO:0046031	ADP metabolic process	0.024101565	0.037969363	0.006903121	HIF1A	1
BP	GO:0090277	positive regulation of peptide hormone secretion	0.024101565	0.037969363	0.006903121	HIF1A	1
BP	GO:0045666	positive regulation of neuron differentiation	0.02436096	0.038179505	0.006941326	NCOA1	1
BP	GO:0045814	negative regulation of gene expression, epigenetic	0.02436096	0.038179505	0.006941326	SIRT1	1
BP	GO:0070301	cellular response to hydrogen peroxide	0.02436096	0.038179505	0.006941326	SIRT1	1
BP	GO:0002793	positive regulation of peptide secretion	0.024620301	0.038387399	0.006979123	HIF1A	1

BP	GO:0060761	negative regulation of response to cytokine stimulus	0.024620301	0.038387399	0.006979123	NR1H3	1
BP	GO:1900542	regulation of purine nucleotide metabolic process	0.024620301	0.038387399	0.006979123	HIF1A	1
BP	GO:0032088	negative regulation of NF-kappaB transcription factor activity	0.024879586	0.038725247	0.007040546	SIRT1	1
BP	GO:0014033	neural crest cell differentiation	0.025138817	0.039061853	0.007101744	HIF1A	1
BP	GO:0014020	primary neural tube formation	0.025657112	0.039596459	0.007198939	HIF1A	1
BP	GO:0032642	regulation of chemokine production	0.025657112	0.039596459	0.007198939	HIF1A	1
BP	GO:1990868	response to chemokine	0.025657112	0.039596459	0.007198939	HIF1A	1
BP	GO:1990869	cellular response to chemokine	0.025657112	0.039596459	0.007198939	HIF1A	1
BP	GO:0006140	regulation of nucleotide metabolic process	0.025916177	0.039793589	0.007234779	HIF1A	1
BP	GO:0030512	negative regulation of transforming growth factor beta receptor signaling pathway	0.025916177	0.039793589	0.007234779	SIRT1	1
BP	GO:0032602	chemokine production	0.025916177	0.039793589	0.007234779	HIF1A	1

BP	GO:0001776	leukocyte homeostasis	0.026175186	0.039921551	0.007258044	HIF1A	1
BP	GO:0002532	production of molecular mediator involved in inflammatory response	0.026175186	0.039921551	0.007258044	HIF1A	1
BP	GO:0006165	nucleoside diphosphate phosphorylation	0.026175186	0.039921551	0.007258044	HIF1A	1
BP	GO:1990830	cellular response to leukemia inhibitory factor	0.026175186	0.039921551	0.007258044	SIRT1	1
BP	GO:1990823	response to leukemia inhibitory factor	0.026434141	0.040248968	0.007317571	SIRT1	1
BP	GO:0008630	intrinsic apoptotic signaling pathway in response to DNA damage	0.02669304	0.040507469	0.007364568	SIRT1	1
BP	GO:0046939	nucleotide phosphorylation	0.02669304	0.040507469	0.007364568	HIF1A	1
BP	GO:0043255	regulation of carbohydrate biosynthetic process	0.026951885	0.040696451	0.007398926	SIRT1	1
BP	GO:0045639	positive regulation of myeloid cell differentiation	0.026951885	0.040696451	0.007398926	HIF1A	1
BP	GO:0070167	regulation of biomineral tissue development	0.026951885	0.040696451	0.007398926	HIF1A	1

BP	GO:0006626	protein targeting to mitochondrion	0.027210674	0.040883476	0.007432929	SREBF1	1
BP	GO:0009135	purine nucleoside diphosphate metabolic process	0.027210674	0.040883476	0.007432929	HIF1A	1
BP	GO:0009179	purine ribonucleoside diphosphate metabolic process	0.027210674	0.040883476	0.007432929	HIF1A	1
BP	GO:0008585	female gonad development	0.027469409	0.041001137	0.007454321	SIRT1	1
BP	GO:0110149	regulation of biomineralization	0.027469409	0.041001137	0.007454321	HIF1A	1
BP	GO:1901655	cellular response to ketone	0.027469409	0.041001137	0.007454321	SIRT1	1
BP	GO:1901796	regulation of signal transduction by p53 class mediator	0.027469409	0.041001137	0.007454321	SIRT1	1
BP	GO:0001841	neural tube formation	0.027728088	0.041251771	0.007499888	HIF1A	1
BP	GO:0022600	digestive system process	0.027728088	0.041251771	0.007499888	NR1H3	1
BP	GO:0002027	regulation of heart rate	0.027986712	0.041365726	0.007520606	SREBF1	1
BP	GO:0010906	regulation of glucose metabolic process	0.027986712	0.041365726	0.007520606	SIRT1	1
BP	GO:0032963	collagen metabolic process	0.027986712	0.041365726	0.007520606	HIF1A	1
BP	GO:0062207	regulation of pattern recognition	0.027986712	0.041365726	0.007520606	NR1H3	1

		receptor signaling pathway					
BP	GO:0006090	pyruvate metabolic process	0.028245281	0.041411227	0.007528878	HIF1A	1
BP	GO:0032526	response to retinoic acid	0.028245281	0.041411227	0.007528878	SREBF1	1
BP	GO:0051341	regulation of oxidoreductase activity	0.028245281	0.041411227	0.007528878	HIF1A	1
BP	GO:0062014	negative regulation of small molecule metabolic process	0.028245281	0.041411227	0.007528878	SIRT1	1
BP	GO:0090398	cellular senescence	0.028245281	0.041411227	0.007528878	SIRT1	1
BP	GO:0042116	macrophage activation	0.028503795	0.041655868	0.007573356	NR1H3	1
BP	GO:0046545	development of primary female sexual characteristics	0.028503795	0.041655868	0.007573356	SIRT1	1
BP	GO:0045833	negative regulation of lipid metabolic process	0.028762255	0.041966115	0.007629761	SIRT1	1
BP	GO:0014066	regulation of phosphatidylinositol 3-kinase signaling	0.029020659	0.042207646	0.007673673	SIRT1	1
BP	GO:0071347	cellular response to interleukin-1	0.029020659	0.042207646	0.007673673	HIF1A	1
BP	GO:0002062	chondrocyte differentiation	0.029795541	0.043127622	0.007840932	HIF1A	1

BP	GO:0009185	ribonucleoside diphosphate metabolic process	0.029795541	0.043127622	0.007840932	HIF1A	1
BP	GO:0009408	response to heat	0.029795541	0.043127622	0.007840932	SIRT1	1
BP	GO:0033559	unsaturated fatty acid metabolic process	0.030053725	0.043432172	0.007896301	SIRT1	1
BP	GO:0002065	columnar/cuboidal epithelial cell differentiation	0.030311854	0.043459741	0.007901314	HIF1A	1
BP	GO:0002832	negative regulation of response to biotic stimulus	0.030311854	0.043459741	0.007901314	NR1H3	1
BP	GO:0030518	intracellular steroid hormone receptor signaling pathway	0.030311854	0.043459741	0.007901314	SIRT1	1
BP	GO:0046916	cellular transition metal ion homeostasis	0.030311854	0.043459741	0.007901314	HIF1A	1
BP	GO:0098586	cellular response to virus	0.030311854	0.043459741	0.007901314	HIF1A	1
BP	GO:0021987	cerebral cortex development	0.030569929	0.043760733	0.007956036	HIF1A	1
BP	GO:0002718	regulation of cytokine production involved in immune response	0.030827948	0.04399153	0.007997997	SIRT1	1
BP	GO:0071346	cellular response to	0.030827948	0.04399153	0.007997997	NR1H3	1

		interferon-gamma					
BP	GO:0002367	cytokine production involved in immune response	0.031343822	0.044518022	0.008093717	SIRT1	1
BP	GO:0002821	positive regulation of adaptive immune response	0.031343822	0.044518022	0.008093717	SIRT1	1
BP	GO:0042752	regulation of circadian rhythm	0.031343822	0.044518022	0.008093717	NR1H3	1
BP	GO:0030278	regulation of ossification	0.031859476	0.045109445	0.008201242	HIF1A	1
BP	GO:0071901	negative regulation of protein serine/threonine kinase activity	0.031859476	0.045109445	0.008201242	SIRT1	1
BP	GO:0002224	toll-like receptor signaling pathway	0.032117221	0.045122957	0.008203699	NR1H3	1
BP	GO:0030282	bone mineralization	0.032117221	0.045122957	0.008203699	HIF1A	1
BP	GO:0046887	positive regulation of hormone secretion	0.032117221	0.045122957	0.008203699	HIF1A	1
BP	GO:0071482	cellular response to light stimulus	0.032117221	0.045122957	0.008203699	SIRT1	1
BP	GO:0072655	establishment of protein localization to mitochondrion	0.032117221	0.045122957	0.008203699	SREBF1	1

BP	GO:0046660	female sex differentiation	0.03237491	0.045414805	0.008256759	SIRT1	1
BP	GO:0043280	positive regulation of cysteine-type endopeptidase activity involved in apoptotic process	0.032632545	0.045705676	0.008309642	SIRT1	1
BP	GO:0003206	cardiac chamber morphogenesis	0.032890125	0.045784263	0.008323929	HIF1A	1
BP	GO:0003231	cardiac ventricle development	0.032890125	0.045784263	0.008323929	HIF1A	1
BP	GO:0006690	icosanoid metabolic process	0.032890125	0.045784263	0.008323929	SIRT1	1
BP	GO:0032411	positive regulation of transporter activity	0.032890125	0.045784263	0.008323929	NR1H3	1
BP	GO:0001838	embryonic epithelial tube formation	0.03314765	0.046072193	0.008376277	HIF1A	1
BP	GO:0030218	erythrocyte differentiation	0.033405121	0.046077776	0.008377292	HIF1A	1
BP	GO:0045471	response to ethanol	0.033405121	0.046077776	0.008377292	SREBF1	1
BP	GO:0045739	positive regulation of DNA repair	0.033405121	0.046077776	0.008377292	SIRT1	1
BP	GO:0046683	response to organophosphorus	0.033405121	0.046077776	0.008377292	SREBF1	1
BP	GO:0070585	protein localization to	0.033405121	0.046077776	0.008377292	SREBF1	1

		mitochondrion					
BP	GO:0048593	camera-type eye morphogenesis	0.033662536	0.046292353	0.008416304	HIF1A	1
BP	GO:0050714	positive regulation of protein secretion	0.033662536	0.046292353	0.008416304	HIF1A	1
BP	GO:0034968	histone lysine methylation	0.033919897	0.04650556	0.008455067	SIRT1	1
BP	GO:0042542	response to hydrogen peroxide	0.033919897	0.04650556	0.008455067	SIRT1	1
BP	GO:0007368	determination of left/right symmetry	0.034177202	0.046787766	0.008506374	HIF1A	1
BP	GO:0009132	nucleoside diphosphate metabolic process	0.034434453	0.046998375	0.008544664	HIF1A	1
BP	GO:0048565	digestive tract development	0.034434453	0.046998375	0.008544664	HIF1A	1
BP	GO:0002702	positive regulation of production of molecular mediator of immune response	0.034691649	0.047278425	0.008595579	SIRT1	1
BP	GO:0043467	regulation of generation of precursor metabolites and energy	0.034948791	0.047557561	0.008646329	HIF1A	1
BP	GO:0007098	centrosome cycle	0.035205877	0.047764391	0.008683932	SIRT1	1
BP	GO:2001235	positive regulation of apoptotic signaling pathway	0.035205877	0.047764391	0.008683932	SIRT1	1

BP	GO:0043401	steroid hormone mediated signaling pathway	0.035462909	0.047969917	0.008721298	SIRT1	1
BP	GO:0071333	cellular response to glucose stimulus	0.035462909	0.047969917	0.008721298	HIF1A	1
BP	GO:0034101	erythrocyte homeostasis	0.035719886	0.04824573	0.008771443	HIF1A	1
BP	GO:0030879	mammary gland development	0.035976808	0.048448768	0.008808357	HIF1A	1
BP	GO:0071331	cellular response to hexose stimulus	0.035976808	0.048448768	0.008808357	HIF1A	1
BP	GO:0006997	nucleus organization	0.036233675	0.048507232	0.008818986	SIRT1	1
BP	GO:0007586	digestion	0.036233675	0.048507232	0.008818986	NR1H3	1
BP	GO:0008203	cholesterol metabolic process	0.036233675	0.048507232	0.008818986	SREBF1	1
BP	GO:0072175	epithelial tube formation	0.036233675	0.048507232	0.008818986	HIF1A	1
BP	GO:0034341	response to interferon-gamma	0.036490488	0.048707567	0.008855409	NR1H3	1
BP	GO:0071326	cellular response to monosaccharide stimulus	0.036490488	0.048707567	0.008855409	HIF1A	1
BP	GO:0009855	determination of bilateral symmetry	0.036747246	0.048906656	0.008891604	HIF1A	1
BP	GO:0070555	response to interleukin-1	0.036747246	0.048906656	0.008891604	HIF1A	1
BP	GO:0009799	specification of symmetry	0.037003949	0.049086787	0.008924354	HIF1A	1

BP	GO:0014074	response to purine-containing compound	0.037003949	0.049086787	0.008924354	SREBF1	1
BP	GO:0010212	response to ionizing radiation	0.037260597	0.049086787	0.008924354	SIRT1	1
BP	GO:0046165	alcohol biosynthetic process	0.037260597	0.049086787	0.008924354	SREBF1	1
BP	GO:0055076	transition metal ion homeostasis	0.037260597	0.049086787	0.008924354	HIF1A	1
BP	GO:0055123	digestive system development	0.037260597	0.049086787	0.008924354	HIF1A	1
BP	GO:2001056	positive regulation of cysteine-type endopeptidase activity	0.037260597	0.049086787	0.008924354	SIRT1	1
BP	GO:0018022	peptidyl-lysine methylation	0.037517191	0.049353294	0.008972807	SIRT1	1
BP	GO:0014065	phosphatidylinositol 3-kinase signaling	0.03777373	0.04961896	0.009021107	SIRT1	1
BP	GO:0017015	regulation of transforming growth factor beta receptor signaling pathway	0.038286643	0.050003677	0.009091051	SIRT1	1
BP	GO:0045598	regulation of fat cell differentiation	0.038286643	0.050003677	0.009091051	SIRT1	1
BP	GO:0106106	cold-induced thermogenesis	0.038286643	0.050003677	0.009091051	NR1H3	1
BP	GO:0120161	regulation of cold-induced	0.038286643	0.050003677	0.009091051	NR1H3	1

		thermogenesis					
BP	GO:0009411	response to UV	0.038799338	0.050383712	0.009160145	SIRT1	1
BP	GO:0061351	neural precursor cell proliferation	0.038799338	0.050383712	0.009160145	HIF1A	1
BP	GO:1902652	secondary alcohol metabolic process	0.038799338	0.050383712	0.009160145	SREBF1	1
BP	GO:2000377	regulation of reactive oxygen species metabolic process	0.038799338	0.050383712	0.009160145	HIF1A	1
BP	GO:0007612	learning	0.039055604	0.05042833	0.009168256	HIF1A	1
BP	GO:0031023	microtubule organizing center organization	0.039055604	0.05042833	0.009168256	SIRT1	1
BP	GO:0034614	cellular response to reactive oxygen species	0.039055604	0.05042833	0.009168256	SIRT1	1
BP	GO:1903844	regulation of cellular response to transforming growth factor beta stimulus	0.039055604	0.05042833	0.009168256	SIRT1	1
BP	GO:0071322	cellular response to carbohydrate stimulus	0.039567971	0.051017426	0.009275359	HIF1A	1
BP	GO:0016331	morphogenesis of embryonic epithelium	0.039824073	0.051274904	0.00932217	HIF1A	1
BP	GO:0007584	response to nutrient	0.040080119	0.051386218	0.009342408	NCOA1	1

BP	GO:0016125	sterol metabolic process	0.040080119	0.051386218	0.009342408	SREBF1	1
BP	GO:0043524	negative regulation of neuron apoptotic process	0.040080119	0.051386218	0.009342408	HIF1A	1
BP	GO:0010675	regulation of cellular carbohydrate metabolic process	0.040336112	0.051496525	0.009362462	SIRT1	1
BP	GO:0016052	carbohydrate catabolic process	0.040336112	0.051496525	0.009362462	HIF1A	1
BP	GO:0035148	tube formation	0.040336112	0.051496525	0.009362462	HIF1A	1
BP	GO:0090101	negative regulation of transmembrane receptor protein serine/threonine kinase signaling pathway	0.041103761	0.052402971	0.009527261	SIRT1	1
BP	GO:0007292	female gamete generation	0.041359535	0.052508124	0.009546379	SIRT1	1
BP	GO:0048592	eye morphogenesis	0.041359535	0.052508124	0.009546379	HIF1A	1
BP	GO:0060041	retina development in camera-type eye	0.041359535	0.052508124	0.009546379	HIF1A	1
BP	GO:0051147	regulation of muscle cell differentiation	0.041615254	0.052685607	0.009578647	SIRT1	1
BP	GO:1990845	adaptive thermogenesis	0.041615254	0.052685607	0.009578647	NR1H3	1

BP	GO:0040029	epigenetic regulation of gene expression	0.042382085	0.053581801	0.009741582	SIRT1	1
BP	GO:0021915	neural tube development	0.042637586	0.053829952	0.009786697	HIF1A	1
BP	GO:2001022	positive regulation of response to DNA damage stimulus	0.043148424	0.054399331	0.009890215	SIRT1	1
BP	GO:0003205	cardiac chamber development	0.043403762	0.054569874	0.009921221	HIF1A	1
BP	GO:0016571	histone methylation	0.043403762	0.054569874	0.009921221	SIRT1	1
BP	GO:0010970	transport along microtubule	0.043659044	0.054815016	0.009965789	HIF1A	1
BP	GO:0001837	epithelial to mesenchymal transition	0.043914273	0.054907942	0.009982684	HIF1A	1
BP	GO:0002262	myeloid cell homeostasis	0.043914273	0.054907942	0.009982684	HIF1A	1
BP	GO:0007568	aging	0.043914273	0.054907942	0.009982684	SREBF1	1
BP	GO:0072331	signal transduction by p53 class mediator	0.044934642	0.056106579	0.010200605	SIRT1	1
BP	GO:0010950	positive regulation of endopeptidase activity	0.045189598	0.056193358	0.010216383	SIRT1	1
BP	GO:0021543	pallium development	0.045189598	0.056193358	0.010216383	HIF1A	1
BP	GO:0031214	biomineral tissue development	0.045189598	0.056193358	0.010216383	HIF1A	1
BP	GO:0010469	regulation of signaling	0.045444499	0.056433128	0.010259975	HIF1A	1

		receptor activity					
BP	GO:0001659	temperature homeostasis	0.046208878	0.057225981	0.010404121	NR1H3	1
BP	GO:0048015	phosphatidylinositol-mediated signaling	0.046208878	0.057225981	0.010404121	SIRT1	1
BP	GO:0048771	tissue remodeling	0.046463561	0.057463098	0.010447231	HIF1A	1
BP	GO:0042770	signal transduction in response to DNA damage	0.046972766	0.057935203	0.010533063	SIRT1	1
BP	GO:0110148	biomineralization	0.046972766	0.057935203	0.010533063	HIF1A	1
BP	GO:0048017	inositol lipid-mediated signaling	0.047227287	0.058170195	0.010575787	SIRT1	1
BP	GO:0050728	negative regulation of inflammatory response	0.047736165	0.058717421	0.010675277	NR1H3	1
BP	GO:0009266	response to temperature stimulus	0.048244826	0.059254918	0.010772998	SIRT1	1
BP	GO:0002221	pattern recognition receptor signaling pathway	0.048499075	0.059254918	0.010772998	NR1H3	1
BP	GO:0002700	regulation of production of molecular mediator of immune response	0.048499075	0.059254918	0.010772998	SIRT1	1
BP	GO:0030324	lung development	0.048499075	0.059254918	0.010772998	SREBF1	1
BP	GO:0048469	cell maturation	0.048499075	0.059254918	0.010772998	HIF1A	1

BP	GO:0006006	glucose metabolic process	0.048753269	0.059405793	0.010800428	SIRT1	1
BP	GO:0030308	negative regulation of cell growth	0.048753269	0.059405793	0.010800428	SIRT1	1
BP	GO:0006839	mitochondrial transport	0.049007409	0.059476282	0.010813243	SREBF1	1
BP	GO:0051896	regulation of protein kinase B signaling	0.049007409	0.059476282	0.010813243	SIRT1	1
BP	GO:0071478	cellular response to radiation	0.049007409	0.059476282	0.010813243	SIRT1	1
BP	GO:0043433	negative regulation of DNA-binding transcription factor activity	0.049261495	0.059625432	0.01084036	SIRT1	1
BP	GO:0050777	negative regulation of immune response	0.049261495	0.059625432	0.01084036	NR1H3	1
BP	GO:0030323	respiratory tube development	0.049515527	0.05985321	0.010881772	SREBF1	1

Table S4 CCs in GO items

ONTOLOGY	ID	Description	pvalue	p.adjust	qvalue	geneID	Count
CC	GO:0090575	RNA polymerase II transcription regulator complex	1.91E-05	0.000400869	0.00016075	NR1H3/NCOA1/HIF1A	3
CC	GO:0000791	euchromatin	8.92E-05	0.000936105	0.000375381	SIRT1/HIF1A	2
CC	GO:0005635	nuclear envelope	0.005731119	0.030088376	0.012065514	SREBF1/SIRT1	2
CC	GO:0005677	chromatin silencing complex	0.003267478	0.022872348	0.009171869	SIRT1	1
CC	GO:1904115	axon cytoplasm	0.015506678	0.047265597	0.018953623	HIF1A	1
CC	GO:0005637	nuclear inner membrane	0.015755199	0.047265597	0.018953623	SIRT1	1
CC	GO:0012507	ER to Golgi transport vesicle membrane	0.015755199	0.047265597	0.018953623	SREBF1	1
CC	GO:0000792	heterochromatin	0.019477004	0.049731002	0.019942257	SIRT1	1
CC	GO:0120111	neuron projection cytoplasm	0.022446334	0.049731002	0.019942257	HIF1A	1
CC	GO:0030134	COPII-coated ER to Golgi transport vesicle	0.02368143	0.049731002	0.019942257	SREBF1	1
CC	GO:0016605	PML body	0.026147876	0.049918672	0.020017513	SIRT1	1
CC	GO:0001650	fibrillar center	0.035963864	0.062936762	0.025237799	SIRT1	1

CC	GO:0030662	coated vesicle membrane	0.048847962	0.078908247	0.031642405	SREBF1	1

Table S5 MFs in GO items

ONTOLOGY	ID	Description	pvalue	p.adjust	qvalue	geneID	Count
MF	GO:0016922	nuclear receptor binding	4.15E-06	0.000199246	2.18E-05	NCOA1/SIRT1/HIF1A	3
MF	GO:0061629	RNA polymerase II-specific DNA-binding transcription factor binding	6.60E-05	0.000931698	0.00010216	NCOA1/SIRT1/HIF1A	3
MF	GO:0001228	DNA-binding transcription activator activity, RNA polymerase II-specific	0.000156573	0.000987896	0.000108322	NR1H3/SREBF1/HIF1A	3
MF	GO:0001216	DNA-binding transcription activator activity	0.000160578	0.000987896	0.000108322	NR1H3/SREBF1/HIF1A	3
MF	GO:0140297	DNA-binding transcription factor binding	0.000164649	0.000987896	0.000108322	NCOA1/SIRT1/HIF1A	3
MF	GO:0004879	nuclear receptor activity	7.76E-05	0.000931698	0.00010216	NR1H3/SREBF1	2
MF	GO:0098531	ligand-activated transcription factor activity	7.76E-05	0.000931698	0.00010216	NR1H3/SREBF1	2
MF	GO:0002039	p53 binding	0.00012925	0.000987896	0.000108322	SIRT1/HIF1A	2
MF	GO:0016410	N-acyltransferase activity	0.000223266	0.001190752	0.000130565	NCOA1/SIRT1	2
MF	GO:0001221	transcription coregulator binding	0.000342514	0.001644068	0.000180271	SREBF1/HIF1A	2
MF	GO:0016747	acyltransferase activity, transferring	0.001397466	0.006098033	0.000668644	NCOA1/SIRT1	2

		groups other than amino-acyl groups					
MF	GO:0016746	acyltransferase activity	0.001755474	0.007021894	0.000769945	NCOA1/SIRT1	2
MF	GO:0003713	transcription coactivator activity	0.002246692	0.008295479	0.000909592	NCOA1/SIRT1	2
MF	GO:0019215	intermediate filament binding	0.003521886	0.012075039	0.001324017	SIRT1	1
MF	GO:0070403	NAD+ binding	0.004333219	0.013866299	0.001520428	SIRT1	1
MF	GO:0004407	histone deacetylase activity	0.005414172	0.016049687	0.001759834	SIRT1	1
MF	GO:0033558	protein lysine deacetylase activity	0.005684264	0.016049687	0.001759834	SIRT1	1
MF	GO:0051019	mitogen-activated protein kinase binding	0.007573264	0.02019537	0.002214405	SIRT1	1
MF	GO:0043425	bHLH transcription factor binding	0.008381956	0.021175467	0.002321871	SIRT1	1
MF	GO:0004402	histone acetyltransferase activity	0.009459391	0.021609738	0.002369489	NCOA1	1
MF	GO:0019213	deacetylase activity	0.009997758	0.021609738	0.002369489	SIRT1	1
MF	GO:0061733	peptide-lysine-N-acetyltransferase activity	0.010266853	0.021609738	0.002369489	NCOA1	1
MF	GO:0030331	nuclear estrogen receptor binding	0.01053589	0.021609738	0.002369489	NCOA1	1
MF	GO:0001223	transcription coactivator binding	0.010804869	0.021609738	0.002369489	HIF1A	1
MF	GO:0051879	Hsp90 protein binding	0.011611453	0.02229399	0.002444516	HIF1A	1

MF	GO:0034212	peptide N-acetyltransferase activity	0.012417511	0.022924636	0.002513666	NCOA1	1
MF	GO:0070888	E-box binding	0.013223044	0.023507633	0.002577591	HIF1A	1
MF	GO:0015485	cholesterol binding	0.013759773	0.023588182	0.002586423	NR1H3	1
MF	GO:0051287	NAD binding	0.014296269	0.0236 6279	0.002594604	SIRT1	1
MF	GO:0030374	nuclear receptor coactivator activity	0.015100575	0.024160919	0.002649224	NCOA1	1
MF	GO:0032934	sterol binding	0.016439918	0.024691268	0.002707376	NR1H3	1
MF	GO:0008080	N-acetyltransferase activity	0.016707611	0.024691268	0.002707376	NCOA1	1
MF	GO:1990841	promoter-specific chromatin binding	0.016975247	0.024691268	0.002707376	SIRT1	1
MF	GO:0016811	hydrolase activity, acting on carbon-nitrogen (but not peptide) bonds, in linear amides	0.018847063	0.026607618	0.002917502	SIRT1	1
MF	GO:0016407	acetyltransferase activity	0.023647215	0.032239205	0.003535	NCOA1	1
MF	GO:0043178	alcohol binding	0.024179403	0.032239205	0.003535	NR1H3	1
MF	GO:0005496	steroid binding	0.027102293	0.035159731	0.003855234	NR1H3	1
MF	GO:0047485	protein N-terminus binding	0.028428561	0.035909762	0.003937474	NCOA1	1
MF	GO:0031490	chromatin DNA binding	0.031605705	0.0388 9933	0.004265277	NR1H3	1
MF	GO:0031072	heat shock protein binding	0.033191157	0.039139396	0.0042 916	HIF1A	1
MF	GO:0016810	hydrolase activity, acting on carbon-nitrogen (but not peptide) bonds	0.034246971	0.039139396	0.0042 916	SIRT1	1

MF	GO:0042826	histone deacetylase binding	0.034246971	0.039139396	0.0042916	HIF1A	1
MF	GO:0008022	protein C-terminus binding	0.048149637	0.053748432	0.005893468	SIRT1	1

Table S6 hub genes in KEGG pathways

ID	Description	pvalue	p.adjust	qvalue	Count
hsa04148	Efferocytosis	5.37E-05	0.001664048	0.000960571	3
hsa04931	Insulin resistance	0.001464663	0.014220113	0.008208554	2
hsa04152	AMPK signaling pathway	0.001834853	0.014220113	0.008208554	2
hsa04919	Thyroid hormone signaling pathway	0.001834853	0.014220113	0.008208554	2
hsa04936	Alcoholic liver disease	0.002517954	0.015463411	0.008926247	2
hsa04932	Non-alcoholic fatty liver disease	0.002992918	0.015463411	0.008926247	2
hsa00760	Nicotinate and nicotinamide metabolism	0.021468127	0.095073134	0.054880926	1
hsa04213	Longevity regulating pathway - multiple species	0.034281793	0.097974479	0.056555726	1
hsa05031	Amphetamine addiction	0.038707118	0.097974479	0.056555726	1
hsa05211	Renal cell carcinoma	0.038707118	0.097974479	0.056555726	1
hsa05230	Central carbon metabolism in cancer	0.039259141	0.097974479	0.056555726	1
hsa03320	PPAR signaling pathway	0.042015454	0.097974479	0.056555726	1
hsa04211	Longevity regulating pathway	0.049699489	0.097974479	0.056555726	1
hsa05235	PD-L1 expression and PD-1 checkpoint pathway in cancer	0.049699489	0.097974479	0.056555726	1

hsa05231	Choline metabolism in cancer	0.054613131	0.097974479	0.056555726	1
hsa04137	Mitophagy - animal	0.057334137	0.097974479	0.056555726	1
hsa04922	Glucagon signaling pathway	0.059506428	0.097974479	0.056555726	1
hsa04066	HIF-1 signaling pathway	0.060048874	0.097974479	0.056555726	1
hsa04659	Th17 cell differentiation	0.060048874	0.097974479	0.056555726	1
hsa04068	FoxO signaling pathway	0.072456228	0.107381542	0.061985949	1
hsa04910	Insulin signaling pathway	0.075671281	0.107381542	0.061985949	1
hsa04915	Estrogen signaling pathway	0.076206256	0.107381542	0.061985949	1
hsa05224	Breast cancer	0.081009887	0.107698754	0.06216906	1
hsa04218	Cellular senescence	0.085793516	0.107698754	0.06216906	1
hsa05160	Hepatitis C	0.086853834	0.107698754	0.06216906	1
hsa04140	Autophagy - animal	0.091613098	0.109231002	0.063053549	1
hsa04310	Wnt signaling pathway	0.095301012	0.10941968	0.063162464	1
hsa05167	Kaposi sarcoma-associated herpesvirus infection	0.105771952	0.117104661	0.067598616	1
hsa05205	Proteoglycans in cancer	0.110452131	0.118069519	0.06815558	1
hsa05208	Chemical carcinogenesis - reactive oxygen species	0.120782385	0.124808465	0.072045633	1
hsa05206	MicroRNAs in cancer	0.164608367	0.164608367	0.09502011	1

Table S7 The TFs associated with hub genes

SYMBOL	TF
SREBF1	CREB1
NCOA1	CREB1
SREBF1	E2F1
SREBF1	EGR1
SIRT1	ESR1
NR1H3	FOXA1
NR1H3	FOXC1
SREBF1	FOXC1
SIRT1	FOXC1
NR1H3	FOXF2
NR1H3	GATA2
HIF1A	GATA2
SREBF1	GATA2
NR1H3	GATA3
HIF1A	GATA3
SREBF1	HINFP
NCOA1	HINFP
SIRT1	HOXA5
SIRT1	MAX
HIF1A	MEF2A
NCOA1	MEF2A
SIRT1	MEF2A
SREBF1	NFIC
SIRT1	NFIC
NR1H3	NFKB1
HIF1A	NFKB1
SREBF1	NFKB1
SIRT1	NFKB1
HIF1A	NKX2-5
HIF1A	NKX3-2

NR1H3	NR2F1
SREBF1	PAX2
NR1H3	PPARG
HIF1A	PPARG
SREBF1	PPARG
HIF1A	RELA
SIRT1	RELA
SREBF1	SIRT1
NR1H3	SRF
SIRT1	SRF
NR1H3	STAT3
SREBF1	STAT3
NCOA1	STAT3
SIRT1	TFAP2A
SIRT1	TFAP2C
gene	TFs
SIRT1	TP63
SIRT1	USF1
SIRT1	USF2
SREBF1	YY1
NCOA1	YY1

Fig S1 The result of TSMR 5 hub genes.

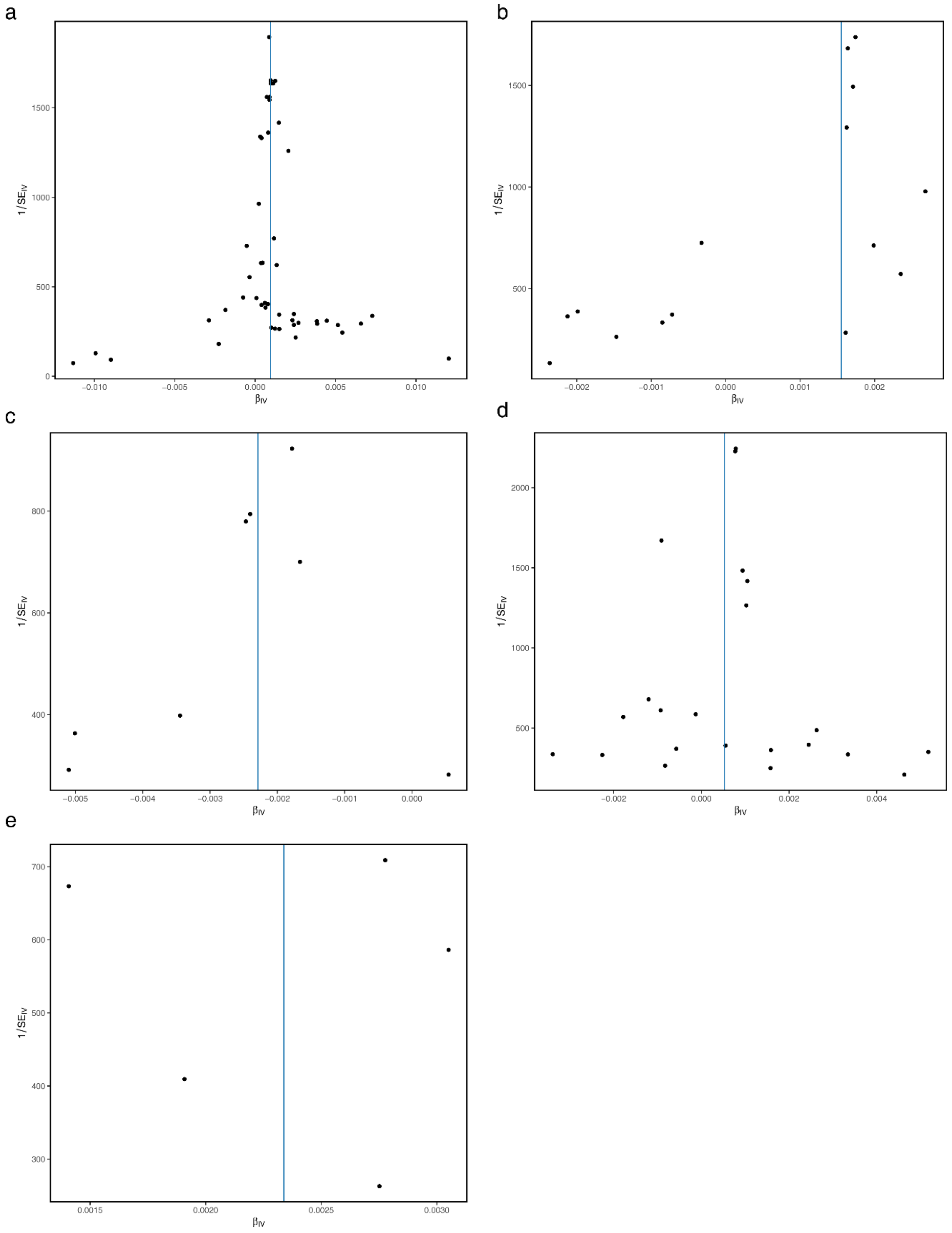

Fig S2 The expression levels of hub genes in immune cells. **A** HIF1A; **B** NCOA1; **C** SREBF1; **D** NR1H3; **E** SIRT1

a

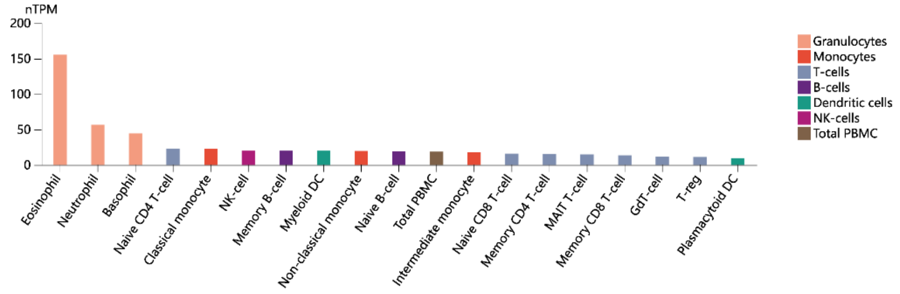

b

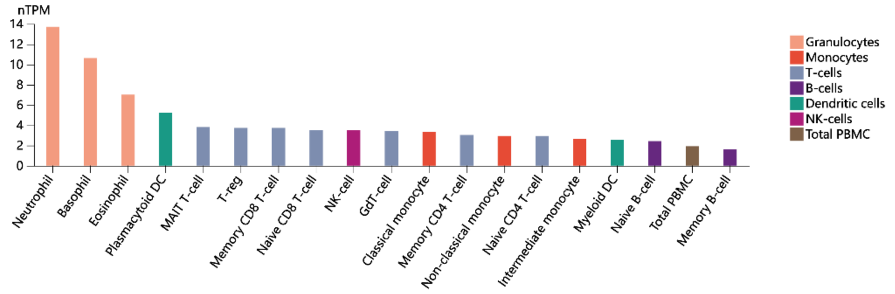

c

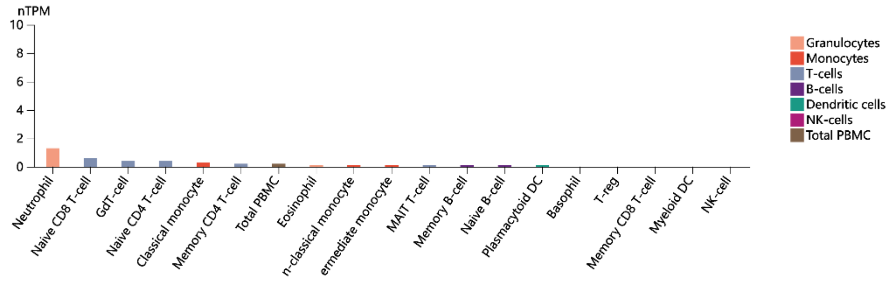

d

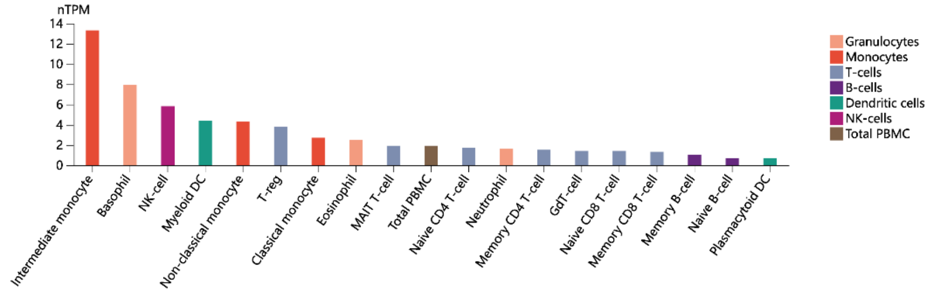

e

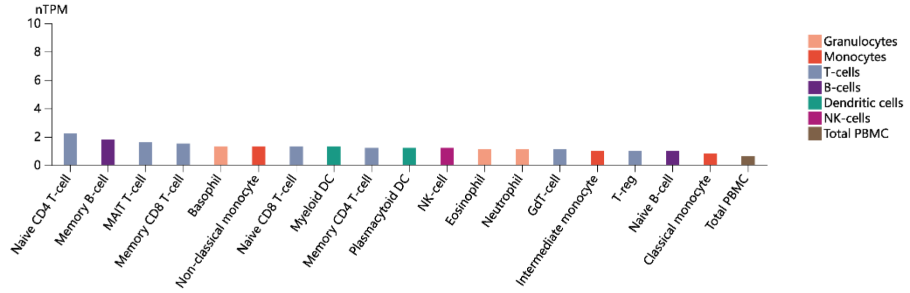

Supplement: Supplementary file 1 [file medi-105-e47228-s001.pdf]
